# Genetic characterization of the first Deltacoronavirus from wild birds around Qinghai Lake

**DOI:** 10.3389/fmicb.2024.1423367

**Published:** 2024-06-12

**Authors:** Ye Tian, Tianqi Yu, Jun Wang, Haoxiang Zhang, Yingna Jian, Xiuping Li, Geping Wang, Guanghua Wang, Yong Hu, Chenhe Lu, Jiyong Zhou, Liqing Ma, Min Liao

**Affiliations:** ^1^Key Laboratory of Animal Virology, Ministry of Agriculture and Rural Affairs, Zhejiang University, Hangzhou, China; ^2^Animal Husbandry and Veterinary Workstation of the Third Division, Xinjiang Production and Construction Corps, Tumushuke, China; ^3^Qinghai Provincial Key Laboratory of Pathogen Diagnosis for Animal Disease and Green Technical Research for Prevention and Control, Qinghai Academy of Animal Sciences and Veterinary Medicine, Qinghai University, Xining, Qinghai, China

**Keywords:** wildbirds, Qinghai Lake, Deltacoronavirus, black-headed gull, receptor-binding domain (RBD)

## Abstract

Deltacoronavirus, widely distributed among pigs and wild birds, pose a significant risk of cross-species transmission, including potential human epidemics. Metagenomic analysis of bird samples from Qinghai Lake, China in 2021 reported the presence of Deltacoronavirus. A specific gene fragment of Deltacoronavirus was detected in fecal samples from wild birds at a positive rate of 5.94% (6/101). Next-generation sequencing (NGS) identified a novel Deltacoronavirus strain, which was closely related to isolates from the United Arab Emirates (2018), China (2022), and Poland (2023). Subsequently the strain was named A/black-headed gull/Qinghai/2021(BHG-QH-2021) upon confirmation of the Cytochrome b gene of black-headed gull in the sample. All available genome sequences of avian Deltacoronavirus, including the newly identified BHG-QH-2021 and 5 representative strains of porcine Deltacoronavirus (PDCoV), were classified according to ICTV criteria. In contrast to *Coronavirus HKU15*, which infects both mammals and birds and shows the possibility of cross-species transmission from bird to mammal host, our analysis revealed that BHG-QH-2021 is classified as *Putative species 4*. *Putative species 4* has been reported to infect 5 species of birds but not mammals, suggesting that cross-species transmission of *Putative species 4* is more prevalent among birds. Recombination analysis traced BHG-QH-2021 origin to dut148cor1 and MW01_1o strains, with MW01_1o contributing the S gene. Surprisingly, SwissModle prediction showed that the optimal template for receptor-binding domain (RBD) of BHG-QH-2021 is derived from the human coronavirus 229E, a member of the Alphacoronavirus, rather than the anticipated RBD structure of PDCoV of Deltacoronavirus. Further molecular docking analysis revealed that substituting the loop 1–2 segments of HCoV-229E significantly enhanced the binding capability of BHG-QH-2021 with human Aminopeptidase N (hAPN), surpassing its native receptor-binding domain (RBD). Most importantly, this finding was further confirmed by co-immunoprecipitation experiment that loop 1–2 segments of HCoV-229E enable BHG-QH-2021 RBD binding to hAPN, indicating that the loop 1–2 segment of the RBD in *Putative species 4* is a probable key determinant for the virus ability to spill over into humans. Our results summarize the phylogenetic relationships among known Deltacoronavirus, reveal an independent putative avian Deltacoronavirus species with inter-continental and inter-species transmission potential, and underscore the importance of continuous surveillance of wildlife Deltacoronavirus.

## 1 Introduction

Coronavirus is a group of enveloped positive-sense single-stranded RNA viruses belonging to the subfamily Orthocoronavirinae within the family Coronaviridae. They harbor the ability to infect mammals and birds, leading to a range of infections and associated symptoms affecting respiratory, gastrointestinal, and neurological systems (Alluwaimi et al., 2020; Zhou et al., 2021). The coronaviruses family is categorized into four genera Alphacoronavirus, Betacoronavirus, Gammacoronavirus, and Deltacoronavirus. SARS-CoV-1 and SARS-CoV-2, both members of the Betacoronavirus genus, from wildlife reservoirs in 2003 and 2019, respectively, resulted in significant global outbreaks, causing substantial loss of life and economic impact (Xiao et al., 2020; Liu et al., 2021). Consequently, coronaviruses represent a group of viruses with a high potential for interspecies transmission, posing significant public health concerns worldwide.

In 2007, Dong et al. (2007) obtained long, unknown viral genomic sequence from Asian leopard cats, characterized by over 12kb of previously unknown viral genomic sequence. Although displaying typical coronavirus genomic features, this virus formed an outgroup phylogenetic relationship with respect to Alphacoronavirus, Betacoronavirus, Gammacoronavirus (Dong et al., 2007). Subsequently, in 2009, Woo et al. detected a similar virus in thrush, bulbuls and munia, and determined their full-length genomic sequences for the first time (Woo et al., 2009). Following confirmation by the International Committee on Taxonomy of Viruses (ICTV), this novel coronavirus was classed into the Deltacoronavirus genus within the subfamily Orthocoronavirinae of the family Coronaviridae. The Deltacoronavirus genus comprises of three subgenera: Andecovirus (including species *Wigeon coronavirus HKU20*), Buldecovirus (including species *Bulbul coronavirus HKU11, Common-moorhen coronavirus HKU21, Coronavirus HKU15*, White-eye coronavirus HKU16) and Herdecovirus (including species *Night-heron coronavirus HKU19*) (Woo et al., 2012; Virus Taxonomy., 2021). In April and June 2018, respectively, Anthony Chamings and Jessy Vibin reported Deltacoronavirus infective wild birds in Australia, with black-headed gulls as hosts (Chamings et al., 2018; Vibin et al., 2018). In addition, Deltacoronavirus was also detected in wild birds in the United States during the same year (Chen et al., 2018). In 2020, Zhu et al. (2021) identified Deltacoronavirus in wildfowl and marmots in Qinghai Province, China, marking the first report of Deltacoronavirus in wildlife within the Qinghai-Tibet Plateau region. Since then, Deltacoronavirus was identified from gulls sequentially in Yunnan Province, China in 2022 and 2023, respectively (Chu et al., 2022; Liao et al., 2023). Experimental evidence indicates that these avian or porcine-derived strains of Deltacoronavirus lack strict host specificity. In 2019, Liang et al. (2019) demonstrated susceptibility of chickens to porcine Deltacoronavirus (PDCoV) infection. In 2023, Liang et al. (2023) reported that pseudoviruses carrying Spike proteins of *Bulbul coronavirus HKU11, Munia coronavirus HKU13*, and Sparrow coronavirus HKU17 could utilize both human and porcine Aminopeptidase N (APN) to invade host cells. Notably, Deltacoronavirus was detected in humans in 2021 (Lednicky et al., 2021).

Case studies mentioned above strongly suggest the cross-species characteristic of strains of Deltacoronavirus and emphasize its importance in public health. Given the abundant nucleotide diversity resulting from mutations and recombinations of coronavirus, the potential for further Deltacoronavirus spillovers into humans should not be underestimated, hence highlighting the importance of investigation into their epidemiological characteristics and summarization of their genomic features. In this study, we investigated the epidemiology of Deltacoronavirus among wild birds around Qinghai Lake in the Qinghai-Tibet Plateau, situated in the eastern part of the plateau, and analyzed its genomics sequences against existing data deposited in GenBank, some of which were classified by ICTV and widely used as references (Virus Taxonomy., 2021). The unique geography and conditions of Qinghai Lake have established it as a natural refuge for migratory birds and a crucial stopover along the migratory pathways of Central Asian and East Asian birds (Cui et al., 2011; Prosser et al., 2011). We identified, for the first time, avian Deltacoronavirus in this region, which exhibited close genetic affinity to a distinct clade (*Putative species 4*) with a broad avian host range.

## 2 Materials and methods

### 2.1 Sample collection and preparation

Sample collection from wild birds around Qinghai Lake was approved by Administration of Qinghai Lake National Nature Reserve. During sample collection, we strictly abided to the rules issued and did not exercise any direct contact with the birds and no interference with the normal activities of wildlife in the area. Fecal samples of wild birds were collected in April 2021 from the Qinghai Lake region. To ensure sample collection from individual birds, fresh fecal samples with uniform color and shape on the ground or glass, instead of those soaked in lake water were collected using new sampling trays, placed in 50 mL centrifuge tubes and transferred to iceboxes within 3 h, followed by storage at −80°C. During the period of sample collection, the overall conditions of wild animals in the patrol area is good, and no abnormal situations have been found.

A small amount of each sample was pooled together and suspended in 50 mL PBS. After three rounds of freeze-thaw, the sample was centrifuged at 13, 400 × g at 4°C for 10 min. The supernatant was subjected to ultracentrifugation at 81, 800 × g at 4°C for 2 h for virus collection, finally the pellet was dissolved in RNase-free water for RNA extraction.

### 2.2 RNA extraction, host determination and Next-generation Sequencing (NGS)

Total RNA was isolated by the Trizol reagent (Vazyme, Nanjing, China) according to the manufacturer's instructions, then subjected to RNA transcription. First-strand cDNA was synthesized using HiScript II First Strand cDNA Synthesis Kit (with gDNA wiper) (Vazyme) with random hexamers, followed by synthesis of second-strand cDNA using Second Strand cDNA Synthesis Kit (Beyotime Biotechnology, Shanghai, China). Purification was performed using AxyPrep DNA Gel Extraction Kit (Axygen, China), and quantification using QuantiFluor-ST Fluorescence Quantitative System (Promega, CA, USA). Following treatment with M220 Focused Ultrasonicator (Covaris Inc. Woburn, MA, USA), DNA was sheared, and 400 bp fragments were excised and extracted. Dual-end library preparation was carried out using TruSeq DNA Sample Preparation Kit (Illumina Inc. San Diego, CA, USA). Paired-end sequencing (150 × 2,500 bp) was then performed on the IlluminaHiSeq 2 system (Illumina Inc. SanDiego, CA, USA).

To determine susceptible host, Cytochrome b (*Cytb*) gene was amplified from the first-strand cDNA from individual sample using mcb primers (mcb398-F: 5′-TACCATGAGGACAAATATCATTCTG-3′; mcb869-R: 5′-CCTCCTAGTTTGTTAGGGATTGATCG-3′) followed by sequencing. Whether belonging to an avian host, sequence identity was queried against online blast analysis (https://blast.ncbi.nlm.nih.gov/Blast.cgi) (Verma and Singh, 2002).

### 2.3 Sequence assembly and analysis

Raw sequence data was quality controlled using FastQC v0.11.9 and trimmed to remove adaptors using Trimmomatic v0.39 (Li et al., 2015). MEGAHIT v1.2.9 (Li et al., 2015) was used to assemble contigs, which were queried against the non-redundant protein database (NR) from NCBI using diamond v2.0.15 (Buchfink et al., 2021) to preliminarily confirm sample composition and design PCR primers for verification.

For samples containing genome sequence of Deltacoronavirus, reads were mapped to reference sequences using bowtie2 v2.4.5 (Langmead and Salzberg, 2012; Langmead et al., 2019) to generate draft genome sequences. Subsequently, sequence assembly and analysis of gap regions were conducted to refine the genome sequences. Downloaded reference sequences were utilized for reference-guided assemblies.

### 2.4 PCR confirmation and amplification of gap regions

Based on conserved regions in Deltacoronavirus sequences deposited in GenBank and the sequence obtained in this study by NGS, one pair of primers for detecting Deltacoronavirus (DCoV-F 5′-CAAAATAATGAATTGTGTTTGCG-3′; DCoV-R 5′-GAACCCATTACTCGTTTAAATAAG-3′) were designed (Table 1). PCR was used to confirm the presence of Deltacoronavirus nucleicacid in the samples collected from wild bird around Qinghai Lake, which was further verified by sequencing.

**Table 1:** Primer list used for PCR confirmation and amplification of gap regions.

**Primer**	**Sequence**	**Length**
DCoV-F	5′-caaaataatgaattgtgtttgcg-3′	760 bp
DCoV-R	5′-gaacccattactcgtttaaataag-3′	
GAP-1-F	5′-actacccacatgttgtgagg-3′	387 bp
GAP-1-R	5′-agtacacgatggctaaagcc-3′	
GAP-2-F	5′-tacagagttgtatccactgg-3′	295 bp
GAP-2-R	5′-tgaccgataacgaacactcc-3′	
GAP-3-F	5′-agtctataaatggttacaacacgg-3′	233 bp
GAP-3-R	5′-attagggttttcaagaacctcc-3′	
GAP-4-F	5′-cgactcactacacttgtatagg-3′	345 bp
GAP-4-R	5′-actactgtttcaactgttatagtagg-3′	
GAP-5-F	5′-gtggtaaccgtaacattgcc-3′	296 bp
GAP-5-R	5′-tgtatcgaagtagtcatttatagattgtacc-3′	
GAP-6-F	5′-ccaccatctgattatgtttacaatcc-3′	270 bp
GAP-6-R	5′-taacaaaatgttaagatagtttgcc-3′	
GAP-7-F	5′-ttgtaatgttgacacataccctg-3′	250 bp
GAP-7-R	5′-agtattacacctagttacacaatctc-3′	
GAP-8-F	5′-tatcaacttgttaatcacttatacaacccag-3′	257 bp
GAP-8-R	5′-ttagtaagtgcttgtacaattgg-3′	
GAP-9-F	5′-ctggcttttgacagaactgg-3′	323 bp
GAP-9-R	5′-gagtggtttgcataaactgg-3′	
GAP-10-F	5′-tgtcacaccctgtagacc-3′	270 bp
GAP-10-R	5′-atgttctgcgtactaatggg-3′	

In order to obtain gap region sequences of the Deltacoronavirus genome, 10 pairs of primers (Table 1) were designed. The expected regions were amplified and sequenced, and then integrated into the draft genome to yield a complete nucleotide sequence of Deltacoronavirus.

### 2.5 Classification of Deltacoronavirus

All the available genome sequences of Deltacoronavirus was downloaded from GenBank (Supplementary Table S1). Due to the widespread circulation of porcine Deltacoronavirus (PDCoV) in pig farms, most of the available genome sequences of Deltacoronavirus in GenBank belongs to PDCoV. Given that large sampling size and single host information would introduce significant bias to the characterization analysis, several representative PDCoV genome sequences were selected, including HKU15–44, HKU15–155, Haiti/human/0081–4/2014, Haiti/human/0256–1/2015, Haiti/human/0329–4/2015 which have been reported to be highly associated with human or wildlife infection events (Woo et al., 2012; Lednicky et al., 2021).

The chosen genome sequences of PDCoV and all available genome sequences of Deltacoronavirus from other species were classified according to the ICTV's classification criteria, namely: based on phylogenetic analysis and calculation of amino acid sequence identity of seven conserved domains ADRP, 3CLpro (nsp5), RdRp (nsp12), Hel (nsp13), ExoN (nsp14), NendoU (nsp15), and O-MT (nsp16) in pp1ab protein, the sequences with 90% amino acid sequence identity were classified into same group.

The classification was visualized using a matrix method. First, the sequences of the seven conserved domains were aligned by MAFFT v7. 505 (Rozewicki et al., 2019) and pairwise similarity of the sequences was calculated by Geneious Prime v2023. 0. 4, followed by plotting Stepped Similarity Heatmap of the above seven conserved domains with 90% similarity as the segmentation standard. Points with similarity above 90% were colored with different depths of dark blue according to numerical size, and points with similarity below 90% were colored with different depths of light blue, with the lightest dark blue clearly darker than the darkest light blue. If a position in the matrix was colored dark blue in the Stepped Similarity Heatmap of the seven conserved domains, this point is defined as a conspecific event and marked in the conspecific matrix. After clustering for conspecific events, a square of marks on the diagonal of the Conspecific matrix with a common diagonal means a conspecific group.

### 2.6 Phylogenetic analysis

Multiple sequence alignment was performed using MAFFT v7. 505 (Rozewicki et al., 2019). The best-fit models and maximum likelihood phylogenetic tree were constructed using IQTREE v2.1.2 (Kalyaanamoorthy et al., 2017; Naser-Khdour et al., 2019; Schrempf et al., 2019), with 10,000 bootstraps of fast iterative optimization.

According to the calculation by IQTREE, the phylogenetic analysis based on the full genome was conducted using GTR+F+R10 model, ADRP domain using LG+G4 model, nsp5 domain using LG+G4 model, nsp12 domain using LG+R3 model, nsp13 domain using LG+I+G4 model, nsp14 domain using LG+R3 model, nsp15 domain using LG+F+G4 model, nsp16 domain sequence using LG+G4 model, RBD domain sequence using WAG+F+G4 model, S gene region using GTR+F+I+G4 model, E gene region using TIM2+F+G4 model, M gene region using GTR+F+G4 model, and N gene region using TIM2+F+I+G4 model.

### 2.7 Analysis of recombination events

Recombination analysis of the full genome was performed using RDP5 v5.46 (Martin et al., 2021) and Simplot v3.5.1 tools, which employed methods including RDP, GENECONV, 3Seq, Chimera, SiScan, MaxChi, BootScan and LARD. Recombination events detected by five or more methods were considered positive. Default parameter values were used for any unspecified parameters.

### 2.8 Prediction and alignment of protein structures

Structures were predicted with the online software SwissModle (https://swissmodel.expasy.org/), accepting only sequences with QMEANDisCo Global scores over 0.5.

Protein tertiary structure alignment employed PyMOL v2.6.0a0 (Schrodinger LLC, 2015) using default parameters.

### 2.9 Molecular docking

Protein-protein molecular docking was conducted using the HDOCK online server (http://hdock.phys.hust.edu.cn/) (Pearson and Lipman, 1988; Berman et al., 2000; Martí-Renom et al., 2000; Larkin et al., 2007; Remmert et al., 2011; Sievers et al., 2011).

Prior to molecular docking, the interaction-active regions of host receptor were determine. Briefly, the demarcation of protein interaction-active regions was conducted using PyMOL. Given that in most cases the maximum effective distance of intermolecular forces is >5 Å (Bissantz et al., 2010), regions corresponding to residues within a 5 Å distance from the ligand molecule were delineated as interaction-active regions.

After molecular docking, salt bridge scanning among protein molecules was performed using PyMOL, following these steps: First, locate all oxygen atoms on Glutamic and Aspartic residues, and all nitrogen atoms on Lysine and Arginine residues. Next, identify salt bridges between located nitrogen atoms and oxygen atoms within a distance >4 Å (Bissantz et al., 2010), and last, manually screen the salt bridges located between molecules.

Hydrogen bond localization was achieved using the built-in functionality of PyMOL. The alignment and calculation of Root Mean Square Deviation (RMSD) for protein tertiary structures were performed using PyMOL with default parameters.

The final output of protein structures is visualized with PyMOL.

### 2.10 Co-immunoprecipitation assays

The gene fragment encoding RBD of 229E isolate DQ243987 was synthesized by Tsingke (Beijing, China), while the gene encoding RBD of A/black-headed gull/Qinghai/2021(BHG-QH-2021) was amplified from samples collected in this study. GFP-QH-RBD, GFP-229E-RBD, GFP-QH-229E-RBD (carrying chimeric loop 1–2 of 229E) vectors were constructed in this study. Flag-hAPN, Flag-pAPN, Flag-gAPN, and Flag-fAPN vectors carrying APN from different host were constructed and kept in the Key Laboratory of Animal Virology, Ministry of Agriculture and Rural Affairs.

293T cells were cultured in DMEM medium containing 10% ExCell serum at 37°C under 5% carbon dioxide concentration. Cotransfection was performed with various APN vectors and RBD vectors using Transfection Reagent (BioBEST) when cells reached 50% confluence, and samples were collected after 36 h. Cell lysates were prepared using NP-40 (Beyotime Biotechnology, Shanghai, China), and FLAG-tagged APN was immunoprecipitated with ANTI-FLAG^®^ M1 Agarose Affinity Gel (Sigma, Shanghai, China). Input original sample and co-immunoprecipitation were then detected by Western blotting using anti-flag and anti-GFP antibodies (HUABIO, Hangzhou, China).

### 2.11 Visualization of bioinformatics analytical results

The results presented in this study were primarily visualized using the ggplot2 package (Wickham, 2016) and treedataverse package in R language. The treedataverse package is a bioinformatics result visualization metapackage created by Professor Yu Guangchuang's research team (Wang et al., 2020; Yu, 2020; Xu et al., 2021a,b).

## 3 Results

### 3.1 Avian strains of Deltacoronavirus present in black-headed gull around Qinghai Lake as revealed by viral metagenomic and RT-PCR

**Figure 1:**
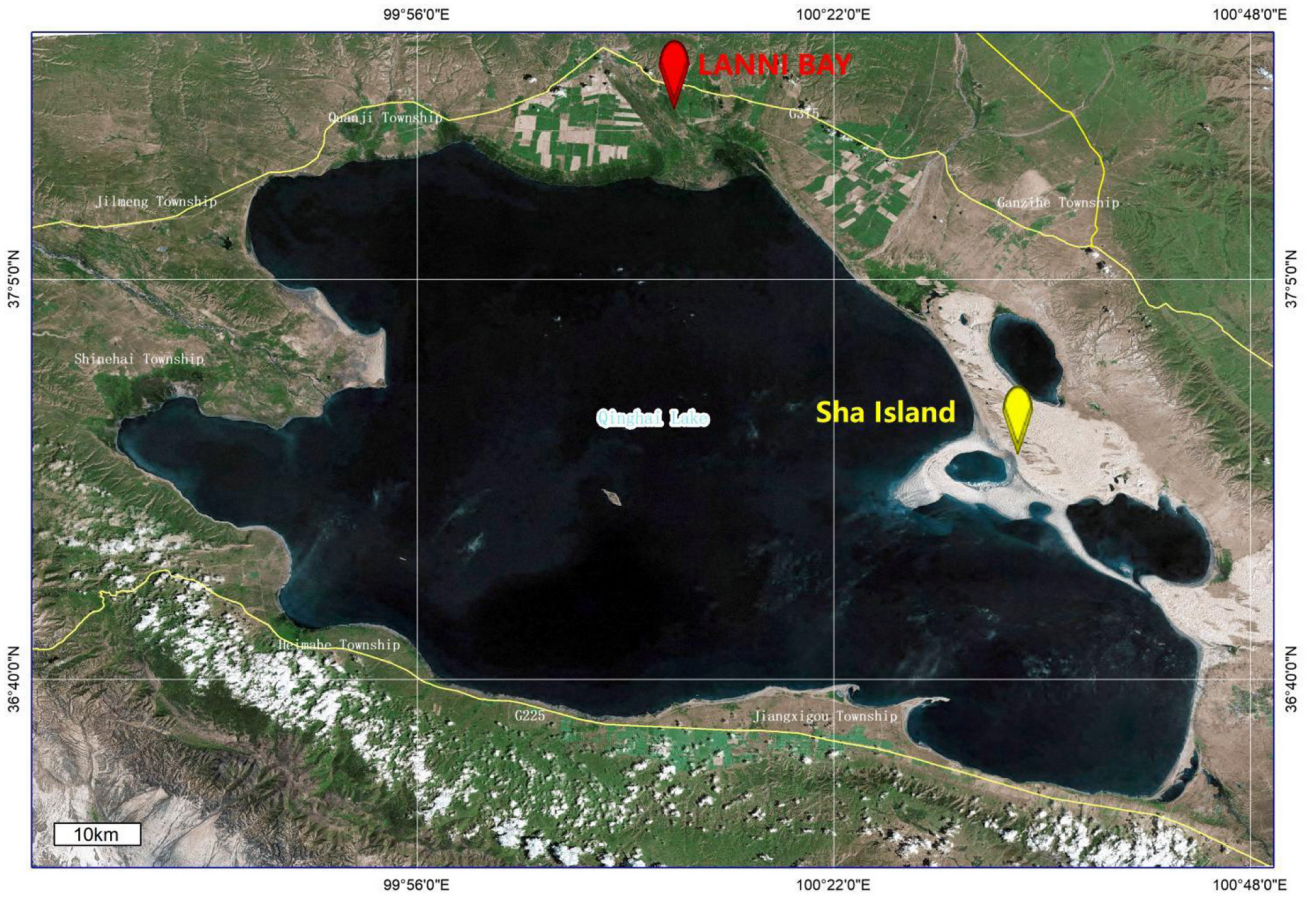
Location of sample collection as marked by ArcGis 10.4.1.

The fecal samples of wild birds in this study were collected from the LANNI BAY and ShaIsland areas around Qinghai Lake (Figure 1). A total of 67 fecal samples were pooled for ultracentrifugation. The RNA extracted from sample pools were subjected to viral metagenomic sequencing using the Illumina platform, employing paired-end reads with a sequencing depth of 2GB, resulting in a total of 1,135,297 reads. Following quality control, assembly and metagenomics analysis of the sequencing data, six contigs (k141_889, k141_1786, k141_1399, k141_4704, k141_6718 and k141_2859) were annotated to Deltacoronavirus.

Based on the obtained contigs sequences and genome sequences of Deltacoronavirus from GenBank, the primer pair (DCoV-F 5′-CAAAATAATGAATTGTGTTTGCG-3′; DCoV-R 5′-GAACCCATTACTCGTTTAAATAAG-3′) was designed for the specific detection of Deltacoronavirus gene fragment from the remains of individual fecal sample. Initially, two out of 7 pools (each containing 10 individual samples with the last pool containing 7 samples) were scored positive by PCR (Supplementary Figure S1A). However, upon individual testing, only samples 53 and 57 collected from LANNI BAY were positive (Supplementary Figure S1B). Using the detection primers, we identified 4 additional positive samples (samples 69, 71, 79, and 87) out of 34 fecal samples collected from the same regions and stored in Trizol, but not submitted to metagenomic sequencing (Supplementary Figure S1C). Therefore, the detection rate of Deltacoronavirus in the samples collected from wild birds in Qinghai Lake was 5.94% (6/101).

Among the positive samples, only the host of sample 57 was successfully identified. The host *Cytb* gene was amplified from sample 57 (Supplementary Figure S2A) and sequenced. Blast search revealed the host identity to be that of a “black-headed gull” (Supplementary Figure S2B). Hence, the Deltacoronavirus strain which infected the black-headed gull (sample 57) and its full genome sequence were thereafter designated “A/black-headed gull/Qinghai/2021 (BHG-QH-2021)”.

**Figure 2:**
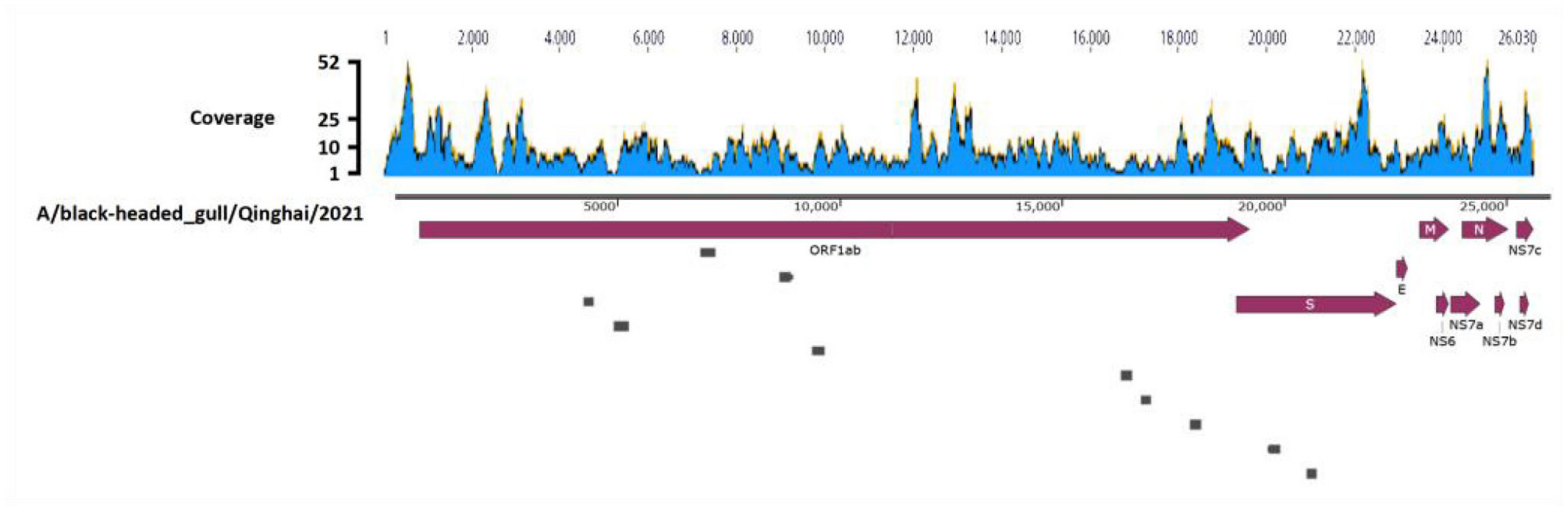
Genome assembly schematic diagram. Diagram illustrates the process of genome assembly. The blue waves, representing Deltacoronavirus genome, indicates the sequence depth of corresponding reads obtained through viral metagenomic sequencing. Dark red thick arrows highlights the annotated encoding protein regions. The small gray boxes represent the ten gaps which were obtained through Sanger sequencing during the assembly process.

Deltacoronavirus positive sample 57 was further subjected to ultracentrifugation for virus purification, RNA extraction and viral metagenomic sequencing, employing paired-end reads with a sequencing depth of 10GB, leading to the generation of 74,114,250 reads. After quality control and annotation, 1,718 reads were annotated gene sequences of Deltacoronavirus, using HNU4–3 as the reference sequence. Sequence depth of the reads was shown in Figure 2 with minimum, maximum, and average sequence depths of the corresponding reads of 3, 52, and 8.97, respectively. The sequences were then assembled with gap regions (Figure 2), which were amplified with the primers listed in Table 1, finally yielding a 25,966 bp viral genome containing all CDS regions of Deltacoronavirus.

### 3.2 Global distribution of avian Deltacoronavirus

All available Deltacoronavirus genome sequences up to June 2023 (Supplementary Table S1) were retrieved from NCBI GenBank (https://www.ncbi.nlm.nih.gov/).To eliminate data bias caused by a large number of PDCoV sequences, we screened and removed most PDCoV sequences, retaining only the five representative PDCoV sequences of HKU15–44, HKU15–155, Haiti/human/0081–4/2014, Haiti/human/0256–1/2015, Haiti/human/0329–4/2015 which have been reported to be highly associated with human or wildlife infection events (Woo et al., 2012; Lednicky et al., 2021). Finally, total of 152 genome sequences including that of BHG-QH-2021 obtained in this study, were subjected for subsequent analysis.

The global distribution of avian Deltacoronavirus was analyzed based on the information of the 136 genomes with clear information on geographic location and host species (Supplementary Table S1, Supplementary Figure S3). We found that avian Deltacoronavirus have been reported in six of the seven continents (including Antarctica) with total of 12 orders of birds as hosts (Supplementary Figure S3) (Chamings et al., 2018; Vibin et al., 2018) Among them, a total of 56 sequences were distributed in Asia, including 27 in China, 13 in Vietnam, 11 in the Republic of Korea and 5 in United Arab Emirates; 52 sequences were distributed in Europe including 26 in Poland, 18 in the Russian Federation, 8 in Italy; and 7 cases in the United States of North America, 12 cases in Australia of Oceania, and 8 cases in Brazil of South America. In terms of the host range infected with avian Deltacoronavirus, Anseriformes were the most frequent host among the 12 orders of bird host species with a total of 30 events reported. Some continents reported fewer host species, for example, only Anseriformes and Charadriiformes were found to be infected with strains of Deltacoronavirus in Oceania, and the virus was only detected in Anseriformes and Passeriformes in North America (Supplementary Figure S3). In Antarctica, wildfowl Deltacoronavirus have been detected in Pygoscelis papua, a species of penguins (Supplementary Figure S3), and the penguins are the dominant bird in Antarctica.

### 3.3 Classification and phylogenetic analysis of Deltacoronavirus

**Figure 3:**
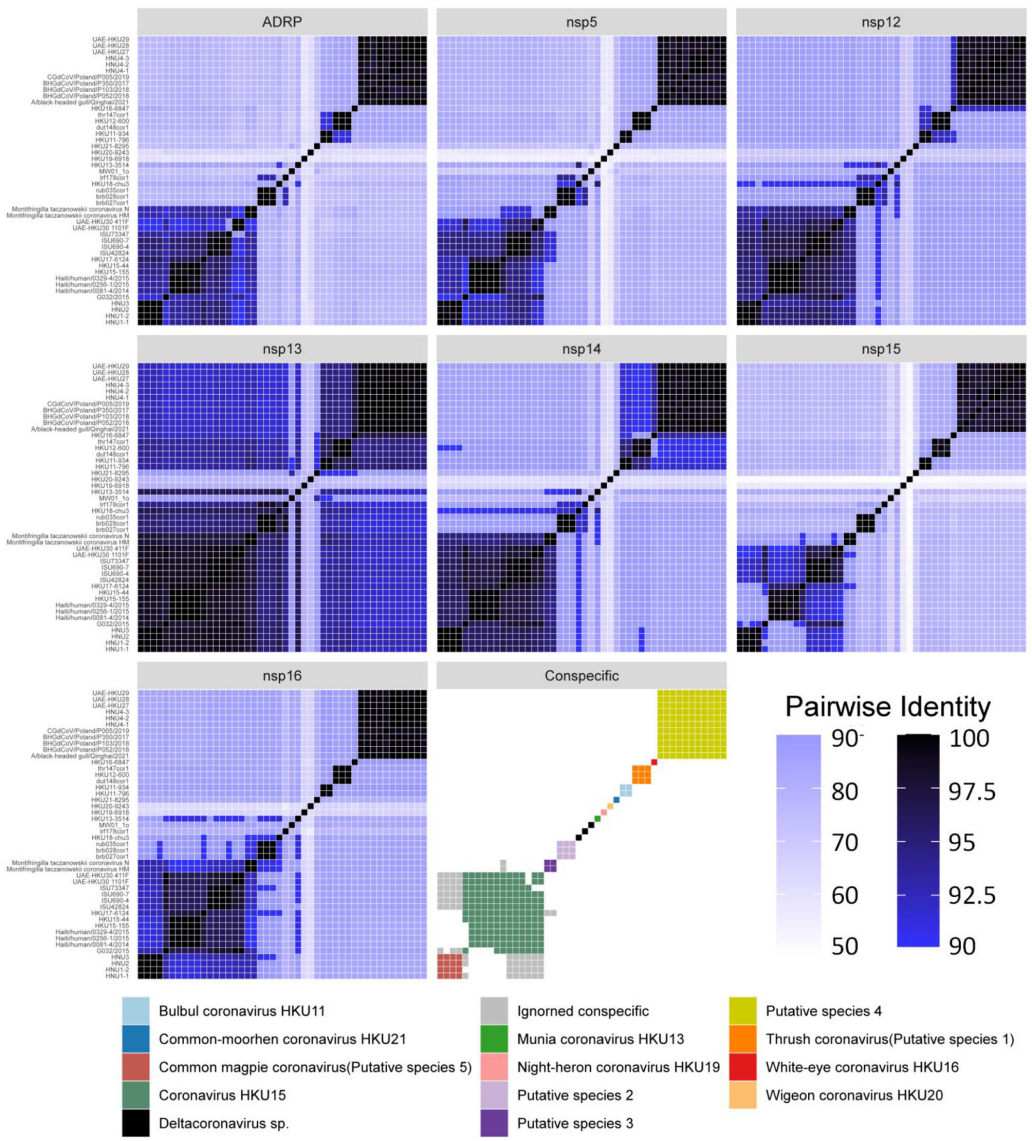
Similarity matrix of Deltacoronavirus species based on conserved domains of 46 full-length sequences. According to the coronavirus classification criteria set by ICTV, sequence identities of seven conserved domains, namely ADRP, 3CLpro (nsp5), RdRp (nsp12), Hel (nsp13), ExoN (nsp14), NendoU (nsp15), and O-MT (nsp16) were calculated. The color gradient from dark to light representing decreasing sequence identity was indicated with intentional separation of more than 90% identity with clearly deep color to emphasize the 90% identity threshold used for species demarcation. The Conspecific matrix is colored to represent the final species classifications.

Among the sequences downloaded from GenBank (Supplementary Table S1), 46 sequences belong to complete genome sequences, including BHG-QH-2021 with complete coding sequences (CDS), which meet the species classification criteria defined by ICTV. Therefore, the 46 full-length genome sequences in the dataset were subjected to classification according to ICTV's classification criteria. The Step Similarity Matrix of Deltacoronavirus species was plotted based on conserved domains (Figure 3).

According to the Conspecific matrix and Phylogenetic tree of 7 conserved domains (Supplementary Figure S4), we confirmed 7 conspecific groups conforming to the species level standards, including 2 known species (*Bulbul coronavirus HKU11* and *Coronavirus HKU15*, previously named by ICTV) and 5 unnamed putative species (*Putative species 1–5*). For species *Common-moorhen coronavirus HKU21, Munia coronavirus HKU13, Night-heron coronavirus HKU19*, White-eye coronavirus HKU16, and *Wigeon coronavirus HKU20* of Deltacoronavirus named by ICTV, we did not find any similar sequences except against their respective reference sequences (HKU21–8295, HKU13–3514, HKU19–6918, HKU16–6847, HKU20–9243) by the Conspecific matrix analysis. The three strains of MW01_1o, HKU18-chu3, and lrf178cor1 could not be classified into any known species and the 5 putative species according to the ICTV's classification criteria, and were conventionally categorized as *Deltacoronavirus sp*.. *Coronavirus HKU15* is the largest species containing a total of 13 identified genome sequences. *Putative species 4* containing BHG-QH-2021 is the second largest species in Deltacoronavirus with 11 genome sequences.

**Figure 4:**
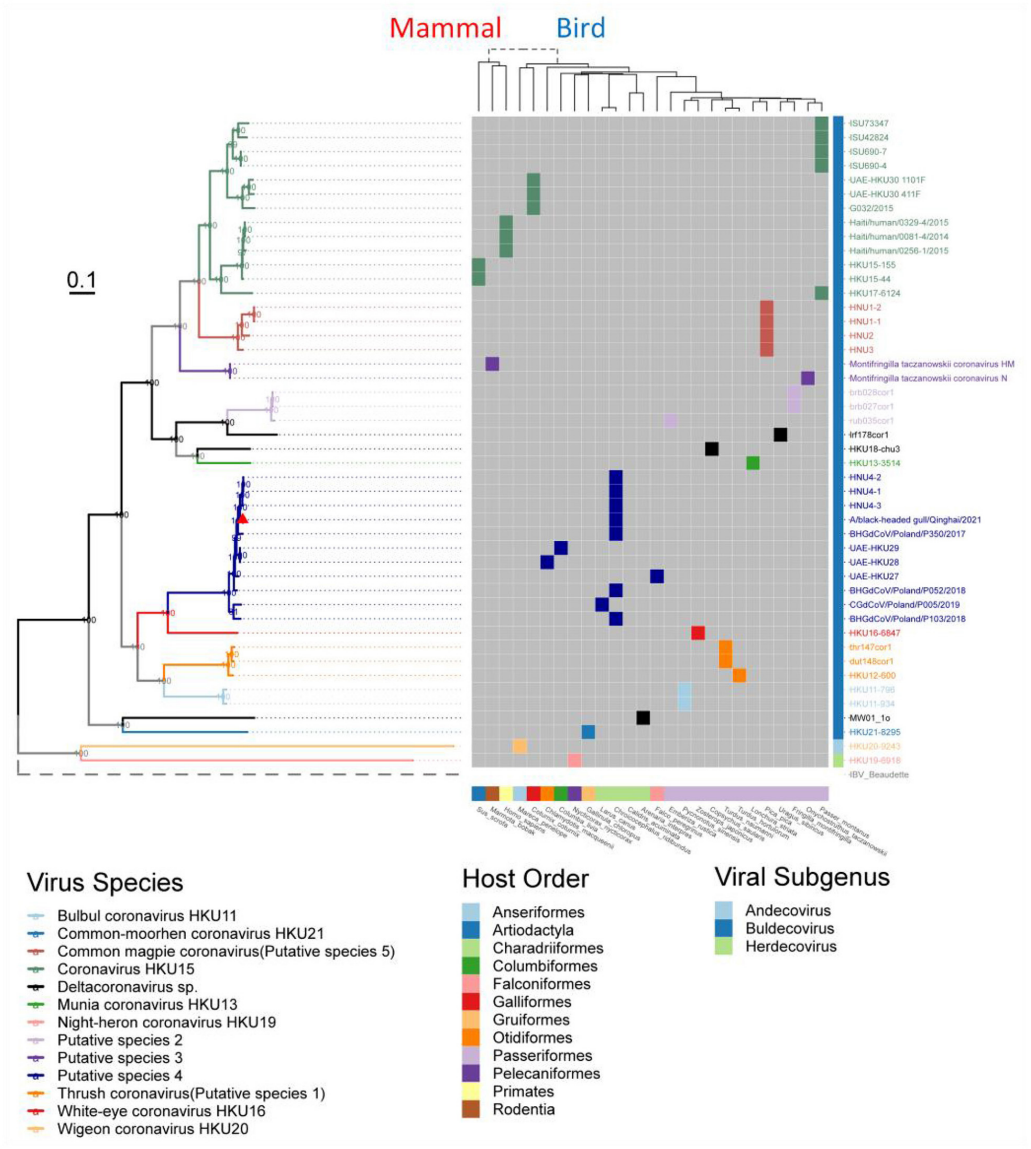
Phylogenetic tree based on the full-length genome sequences of Deltacoronavirus and its host phylogenetic relationship. The genome phylogenetic tree with all nodal support values above 90% was constructed using the maximum likelihood method with IBV reference strain Beaudetteas as an outgroup. Viral species are classified using different colors, with the strain A/black-headed gull/Qinghai/2021 identified in this study indicated by a red triangle. The host evolution tree showing different species classifications was generated using the online tool TimeTree. Virus names and taxonomic classifications are referred to ICTV, while host names and classifications are referred to NCBI taxonomy and WiKi information.

We classified the genus Deltacoronavirus according to the classification criteria of ICTV, but encluded two events that they did not meet the 90% amino acid sequence identity criteria. UAE-HKU30 411F and ISU73347 exhibit 89.68% and 89.68% identities in NendoU (nsp15) domain with Haiti/human/0081–4/2014, Haiti/human/0256–1/2015 Haiti/human/0329–4/2015, but met the 90% amino acid sequence identity criteria in the other 6 domains except NendoU domain, therefore, UAE-HKU30 411F and ISU73347 strains together with Haiti/human/0081–4/2014, Haiti/human/0256–1/2015 Haiti/human/0329–4/2015 strains were classified into the same species *Coronavirus HKU15* (Figure 3). And the classification results were supported by the topological structures of both conserved domains (Supplementary Figure S4) and genome evolutionary trees (Figure 4).

In addition, we excluded two events that could meet the conspecific requirements: First, HKU17–6124 met the conspecific requirement of both members of *Coronavirus HKU15* and *Putative species 3* (Figure 3). Considering that the topologies of genome phylogenetic trees supporting HKU17–6124 belonging to Coronavirus HKU15, therefore, HKU17–6124 was assigned to *Coronavirus HKU15* species (Figure 3); Second, the avian-sourced strains of Coronavirus HKU15 including UAE-HKU30 411F, UAE-HKU30 1101F, ISU690–4, ISU690–7, ISU42824, ISU73347, G032/2015 but not the above mentioned HKU17–6124 showed close conspecific relationship with crow-sourced strains in *Common magpie coronavirus (Putative species 5)* (Figure 3), while in the topological structures of conserved domains (Supplementary Figure S4) and genome evolutionary trees (Figure 4), they were mainly clustered with mammal-sourced *Coronavirus HKU15*, so the avian-sourced strains were assigned to *Coronavirus HKU15*, and *Common magpie coronavirus (Putative species 5)* were thought as a *Coronavirus HKU15*-related species.

To understand the evolution of avian Deltacoronavirus and its relationship with virus subgenus/species and host species, we first constructed a maximum likelihood phylogenetic tree based on 46 full-length Deltacoronavirus genomes, with Gammacoronavirus IBV reference Beaudette strain as an outgroup (Figure 4). In the phylogenetic tree, all virus species with multiple strains (including 2 known species and 5 putative species) were clustered into distinct and separate branches, reflecting their independent evolutionary lineages. *Coronavirus HKU15* species was clustered into the branch colored by green, which is most closely related to *Common_magpie coronavirus (Putative species 5)* species viruses. All the strains in *Putative species 4* were clustered into the dark blue branch, including BHG-QH-2021 and strains HNU4–1, HNU4–2, and HNU4–3, which were identified in the Yunnan region of China (Chu et al., 2022). This branch is closely to *White–eye coronavirus HKU16*.

Except for the topological relationship between *Coronavirus HKU15* and *Common_magpie coronavirus (Putative species 5)*, the cluster of all species in the phylogenetic analysis of domains in ORF1ab (Supplementary Figure S4) was consistent with that of the full-length genome sequence (Figure 4). BHG-QH-2021 is clustered with *Putative species 4* in all evolutionary trees.

In comparison to that based on the conserved domains, the phylogenetic trees based on the coding sequences of structural proteins S, E, M, N, especially the S protein, reflect a stronger association with the pathogenicity of the virus (Kumavath et al., 2021). Therefore, we reconstructed phylogenetic tree based on S, E, M, and N proteins (Supplementary Figure S5), and the reconstruction showed that the classification for Deltacoronavirus (Figure 3) yield good clustering characteristics for proteins E, M, and N (Supplementary Figure S5).

Interestingly, it is observed that for the S protein (Supplementary Figure S5), the strains of *Common_magpie coronavirus (Putative species 5)* did not form an independent branch as that in the phylogenetic trees based on full-length genome, conserved domains, and the remaining structural proteins M, N and E. In those trees, HNU3 is clustered with *Bulbul coronavirus HKU11*, HNU2 clung with White-eye coronavirus HKU16, and HNU1–1 and HNU1–2 clustered with *Thrush coronavirus (Putative species 1)*. Meanwhile, HKU17–6124 of *Coronavirus HKU15* is clustered with *Putative species 3*, rather than *Coronavirus HKU15* (Supplementary Figure S5).

**Figure 5:**
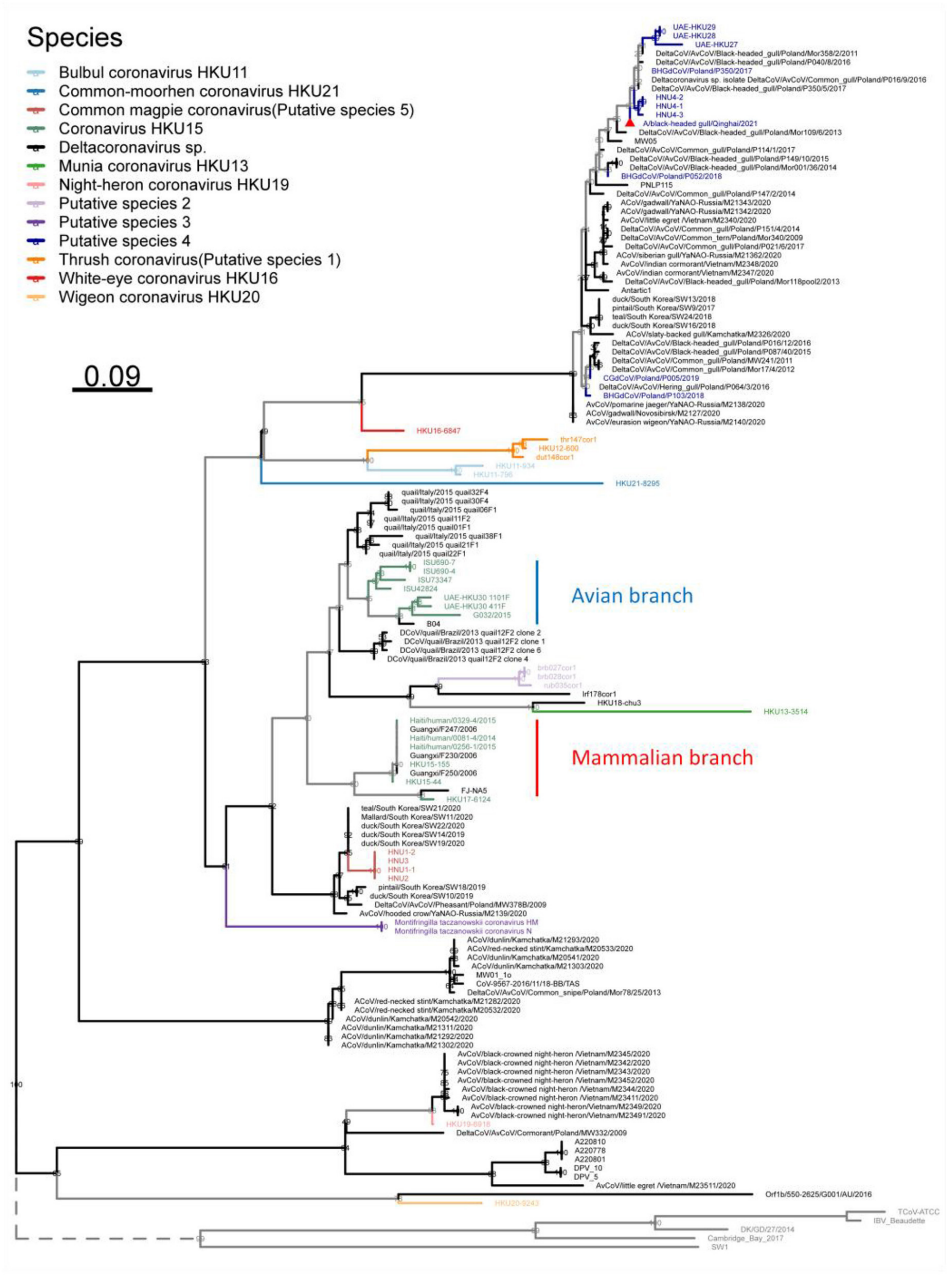
Phylogenetic tree based on a 217 nucleotides fragment encoding nsp12 of 135 Deltacoronavirus sequences using the maximum likelihood method. Gammacoronavirus species representative strains were used as outgroups with their branch lengths omitted and indicated by dashed lines. Viral species are classified using different colors, with the novel strain A/black-headed gull/Qinghai/2021 detected in this study denoted by a red triangle.

Furthermore, in order to analyze the phylogenetic evolution of Deltacoronavirus with more sequences, we first screened for the gene fragments most suitable for phylogenetic tree building. Considering that nsp12 is an essential replicase gene of Coronavirus and is widely used for virus identification (Wu et al., 2020) and gene barcode studies of Alphacoronavirus and Betacoronavirus (Nemr and Radwan, 2022), a suitable fragment length of 217 nucleotides of nsp12 gene was selected. Finally, a maximum likelihood phylogenetic tree was constructed based on the 217nt-nsp12 of 135 Deltacoronavirus sequences, with the corresponding gene fragments of 5 representative strains of Gammacoronavirus as an outgroup (Figure 5). In the phylogenetic analysis, we made a surprising discovery that the *Putative species 2* and *Munia coronavirus HKU13* were inserted into the branch of *Coronavirus HKU15*, leading to the strains in *Coronavirus HKU15* being divided into two parts: the avian branch and the mammalian branch, with the exception that avian strain HKU17–6124 was classified into a mammalian branch (Figure 5). This novel discovery renews our current understanding of virus lineages and highlights the complex dynamics of cross species transmission of Coronavirus.

### 3.4 Recombination in A/black-headed gull/Qinghai/2021

**Figure 6:**
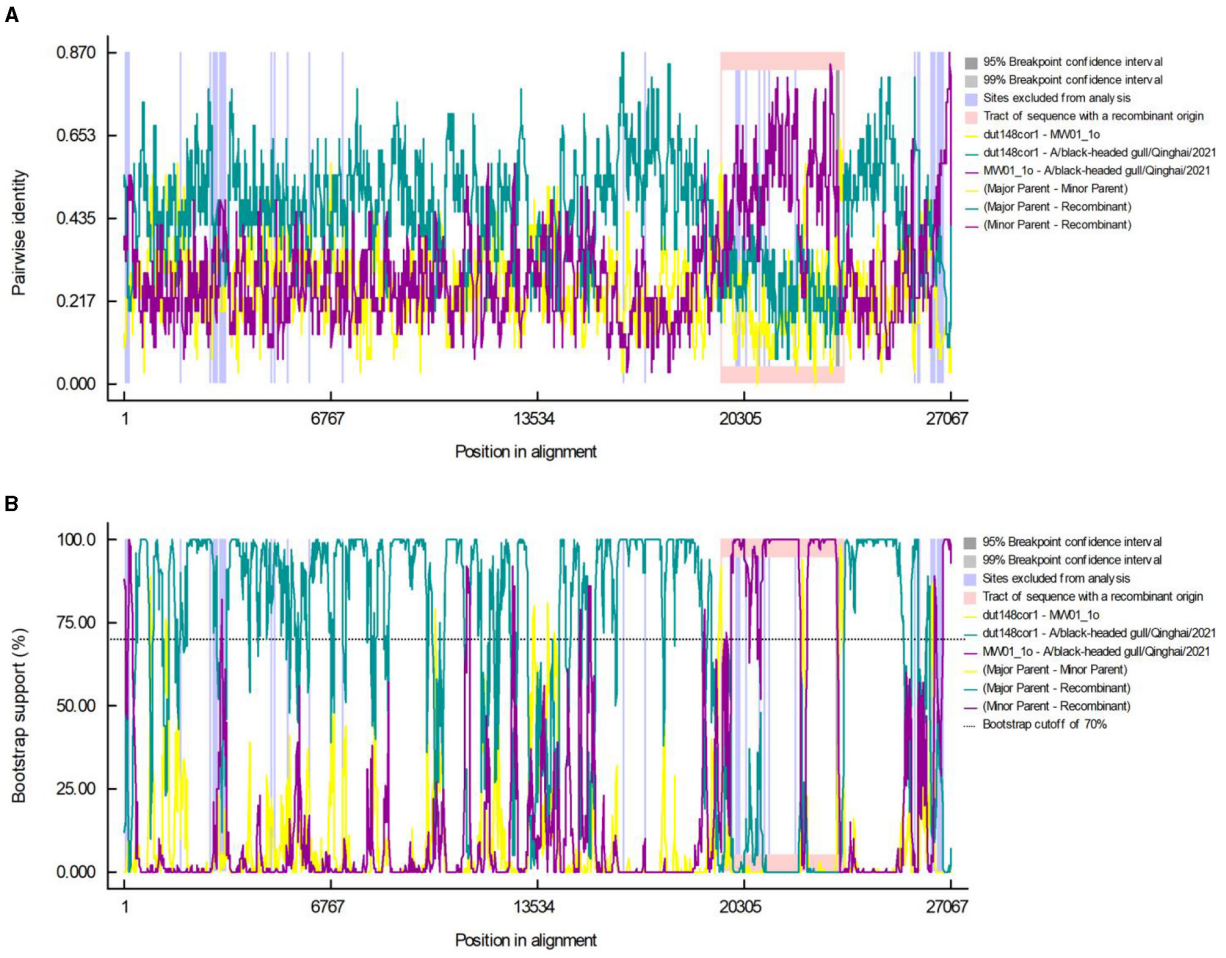
Recombination events in the A/black-headed gull/Qinghai/2021 genome. Pairwise identity **(A)** and bootstrap support **(B)** results calculated by RDP are shown.

Using the RDP5 software, we performed recombination events analysis within Deltacoronavirus genomes. Following screening, two distinct recombination breakpoints were detected in strain BHG-QH-2021 at positions 19,519^th^ nucleotide and 23,616^th^ nucleotide, spanning the entire S gene region. The major and minor parental strains were determined to be dut148cor1 of *Thrush coronavirus (Putative species 1)* and MW01_1o of *Deltacoronavirus sp*., respectively (Figures 6A, B).

**Figure 7:**
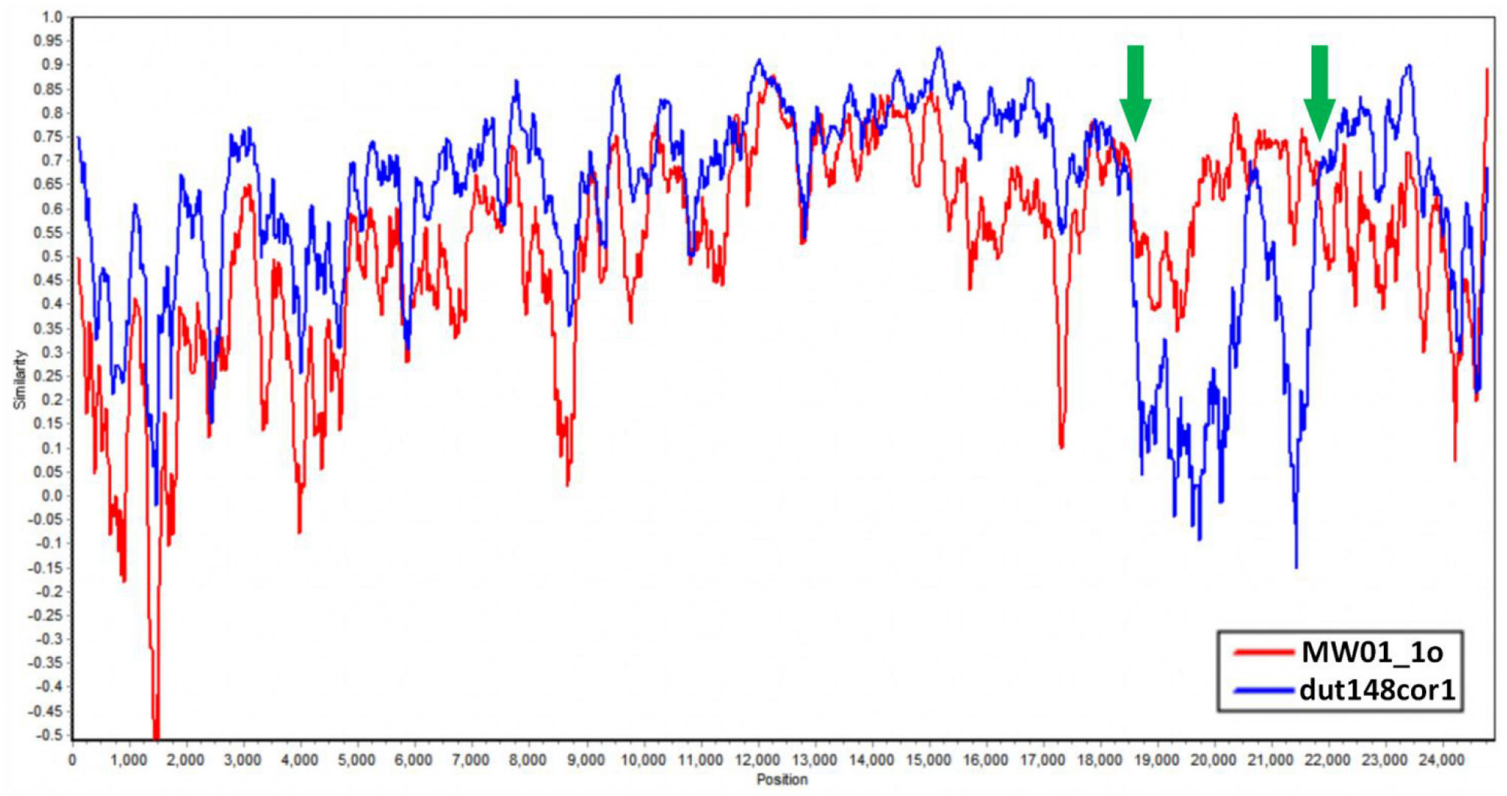
Similarity plot of *Putative species 4* strains compared to dut148cor1 and MW01_1o, calculated and plotted using Simplot v3. 5. 1. Green arrows show the crossover points.

We aim to determine whether the recombination observed in BHG-QH-2021 can be extended to the strains within *Putative species 4*. To validate this speculation, we used Simplot to plot the similarity curve between dut148cor1, MW01_1o and the strains of *Putative species 4* (Figure 7). We identified distinct curve crossover points in the upstream and downstream regions of the S gene coding sequence shown by the green arrows in Figure 7. In previous phylogenetic analysis, *Putative species 4* were clustered with MW01_1o in the same branch with 100% nodal support only in the phylogenetic tree based on S gene, while kept separated in the remaining phylogenetic trees based on complete genome (Figure 4), strongly indicating a close evolutionary relationship between the S genes of *Putative species 4* strains and MW01_1o.

Based on the above data, we put forward the conclusion that the strains in *Putative species 4* originated through recombination between dut148cor1 and MW01_1o, with MW01_1o providing the S gene sequence.

### 3.5 Structural analysis of the receptor-binding domain of Deltacoronavirus

**Figure 8:**
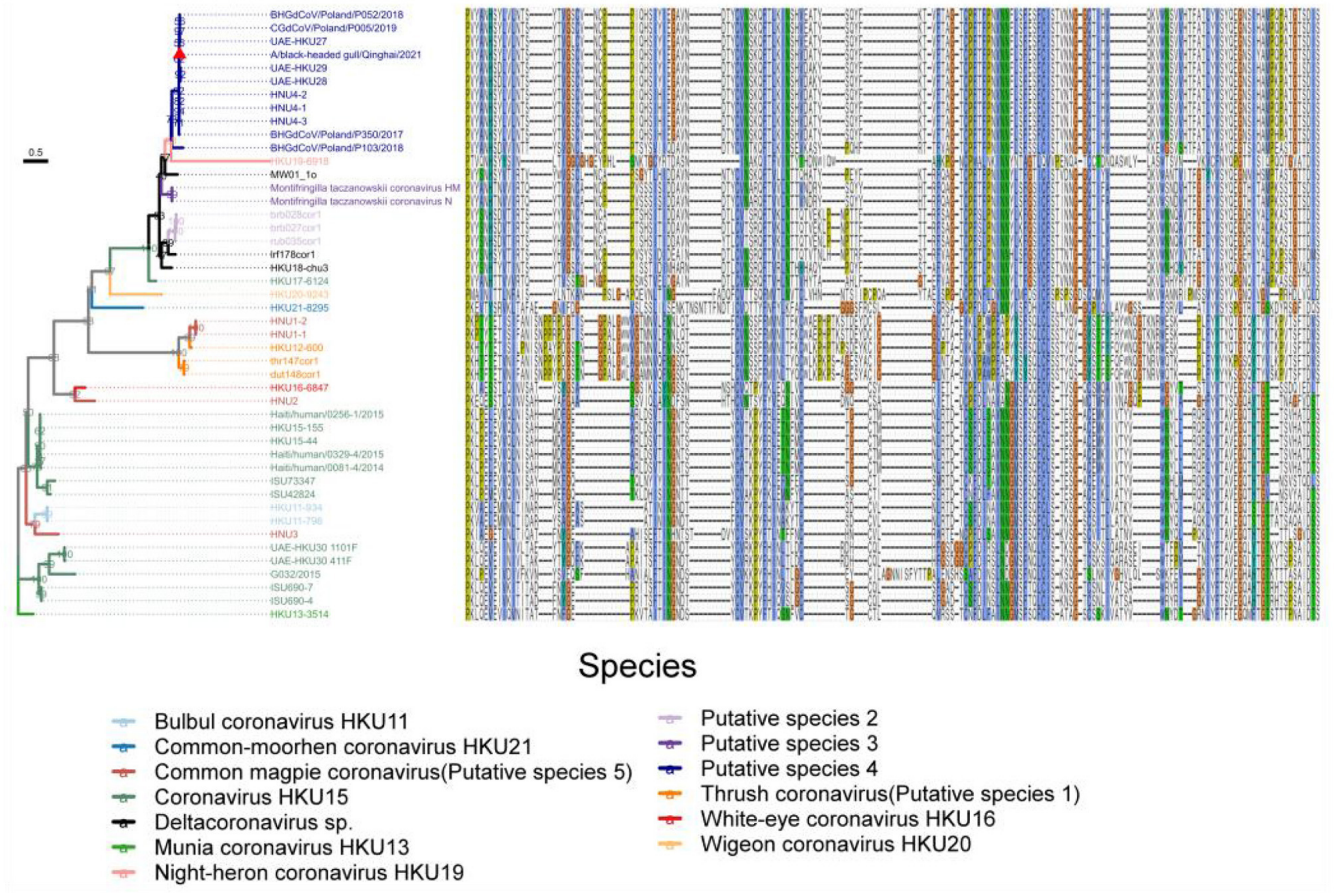
Maximum likelihood phylogenetic tree based on the S gene coding sequences of 46 full-length genome sequences of Deltacoronavirus. Amino acid sequence plots of the corresponding RBD are shown on the right. Viral species are classified using different colors and the strain A/black-headed gull/Qinghai/2021 detected in this study was indicated by a red triangle.

Based on the knowledge of receptor-binding domain (RBD) of PDCoV, we predicted the location of RBD of Deltacoronavirus, and integrated the alignment of RBD sequences of different strains alongside the maximum likelihood phylogenetic tree based on the S gene coding sequence, to construct an association map (Figure 8). Our analysis revealed a consistent correspondence between the patterns of RBD sequence alignments and the phylogenetic relationships observed within the S gene coding sequences. Notably, the RBD profiles exhibited significant similarity among strains within each phylogenetic branch. Although HKU19–6918 clusters with *Putative species 4* in the phylogenetic relationship of the S protein, the RBD sequence profiles revealed that the sequences of *Putative species 2, Putative species 3*, along with *Deltacoronavirus sp*. strains MW01_1o, lrf178cor1, and HKU18-chu3, are more similar to *Putative species 4*. This finding underscores the importance of including RBD sequence data alongside phylogenetic analysis for a comprehensive understanding of viral evolution. Furthermore, the RBD sequence profiles demonstrate a high degree of sequence conservation between *Common_magpie coronavirus (Putative species 5)* strains HNU1–1 and HNU1–2 with *Thrush coronavirus (Putative species 1)*, while exhibiting distinct variations from the profiles of the other two strains of *Common_magpie coronavirus (Putative species 5)*, HNU2 and HNU3. This observation is consistent with the clustering relationship of *Common_magpie coronavirus (Putative species 5)* in the S protein phylogenetic analysis.

To explore the structural relationships of Deltacoronavirus RBDs, we utilized SwissModle to identify a template that closely matches the RBD of strain BHG-QH-2021 for three-dimensional modeling. Surprisingly, the optimal template was derived from the human coronavirus 229E, a member of the Alphacoronavirus genus (QMEANDisCo=0.64, RCSB:6U7G), rather than the RBD structure of PDCoV, although both viruses utilize aminopeptidase N (APN) as their functional receptor.

We aligned the RBD sequences of Deltacoronavirus with those of 229E RBD using Mafft, excluding sequences with less than 30% coverage of the 229E sequence. Among the remaining sequences were strains representing *Putative species 2, Putative species 3, Putative species 4, Common-moorhen coronavirus HKU21, Wigeon coronavirus HKU20*, HKU17–6124 strain of *Coronavirus HKU15*, and *Deltacoronavirus sp*. strains HKU18-chu3, lrf178cor1, MW01_1o. These sequences exhibited pairing similarities ranging from 21.23%-25.87% compared to that of 229E. Notably, except for the RBD sequences of strains from *Common-moorhen coronavirus HKU21* and *Wigeon coronavirus HKU20*, the RBDs of the remaining strains formed distinct clusters in the phylogenetic tree based on the S gene (Figure 8). We designated these clusters as “229E-related Deltacoronavirus RBDs.”

**Figure 9:**
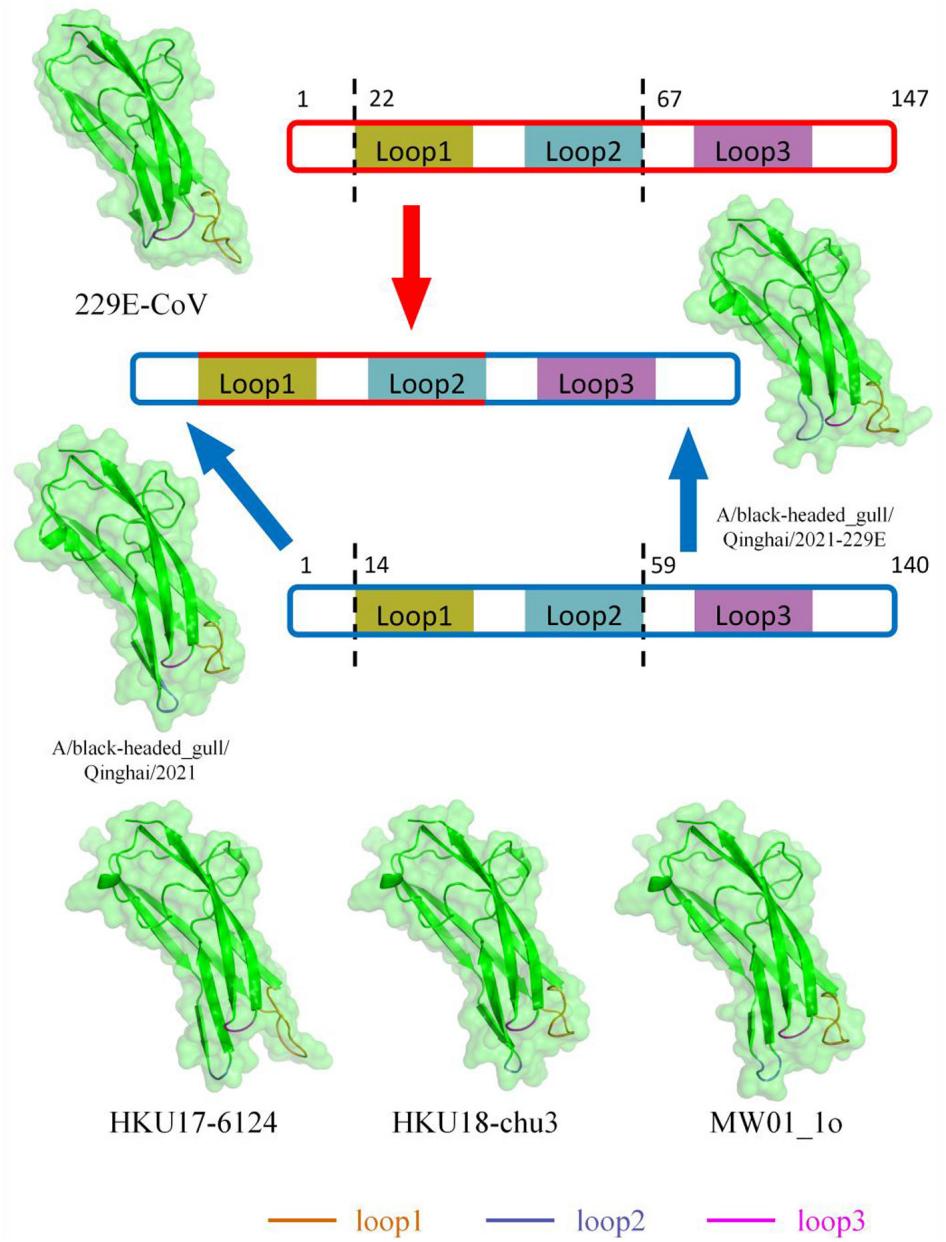
The structures of RBD of 229E-related Deltacoronavirus which was reconstructed using SwissModel with RCSB:6U7G as a template through homology modeling.

Based on previous structural studies of the 229E RBD, the receptor-binding loops of 229E are crucial structures for RBD binding to receptors (Li et al., 2019). According to homology modeling results, the loop 1 of BHG-QH-2021 is less protruding compared to 229E, while loop 2 bulged out more (Figure 9). Homology modeling was also conducted for HKU17–6124, HKU18-chu3, and MW01_1o within “229E-related Deltacoronavirus RBDs,” representing a mammalian-related Deltacoronavirus, the Deltacoronavirus with the highest similarity (25.87%) to the 229E RBD, and the minor parental strain of *Putative species 4*, respectively. The loop 1 and loop 2 of the three strains exhibit characteristics similar to those of BHG-QH-2021 when compared to 229E (Figure 9). We transferred the loop 1–2 region of 229E RBD to the RBD of BHG-QH-2021, named BHG-QH-2021–229E. Homology modeling revealed that the structure of its receptor-binding loops resembled those of the RBD of 229E to a greater extent (Figure 9).

### 3.6 Interaction between APN and RBD of 229E-related Deltacoronavirus

**Figure 10:**
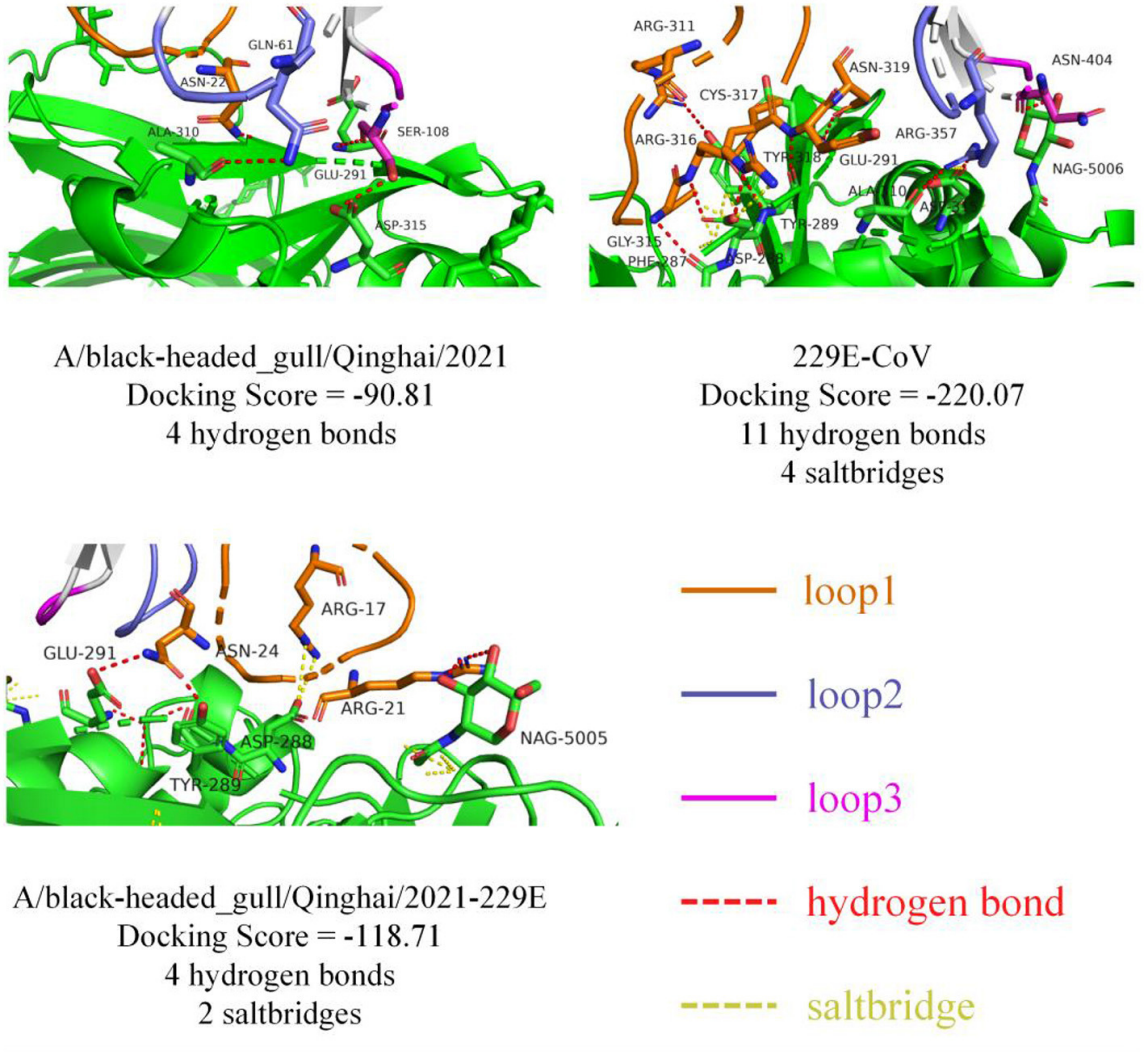
The molecular docking results of 229E RBD, A/black-headed gull/Qinghai/2021 RBD, and A/black-headed gull/Qinghai/2021–229E RBD with hAPN are depicted, with hydrogen bonds and salt bridges represented by red and yellow dashed lines, respectively.

To probe the structural relevance of the aforementioned structural features on RBD binding to APN, we conducted molecular docking between RBD and human APN (hAPN). Firstly, we defined the interaction-active regions of hAPN based on the reference structure 6U7G (241–244 aa, 285–293 aa, 309–312 aa, free aa 5006), and set a maximum distance of 8Å between the receptor-binding loops of RBD and hAPN. Subsequently, molecular docking was performed. The docking results between 229E RBD and hAPN were identical to those in 6U7G (RMSD=0.000), validating the feasibility of the docking method. The degree of molecular binding was represented by docking scores, where smaller values indicate tighter binding. The docking score between 229E RBD and hAPN was −220.07, with 11 hydrogen bonds and 4 salt bridges identified between the molecules. For BHG-QH-2021 and hAPN, the docking score was −90.81, with 4 hydrogen bonds identified (Figure 10). Meanwhile, for the complex of BHG-QH-2021–229E and hAPN, the docking score was −118.71, with 4 hydrogen bonds and 2 salt bridges identified at the molecular interface. Additionally, the docking scores of HKU17–6124, HKU18-chu3, and MW01_1o RBDs with hAPN were −114.13, −104.50, and −96.99, respectively, indicating that the binding of BHG-QH-2021–229E RBD to hAPN is stronger than those of HKU17–6124, HKU18-chu3, and MW01_1o RBDs to hANP.

**Figure 11:**
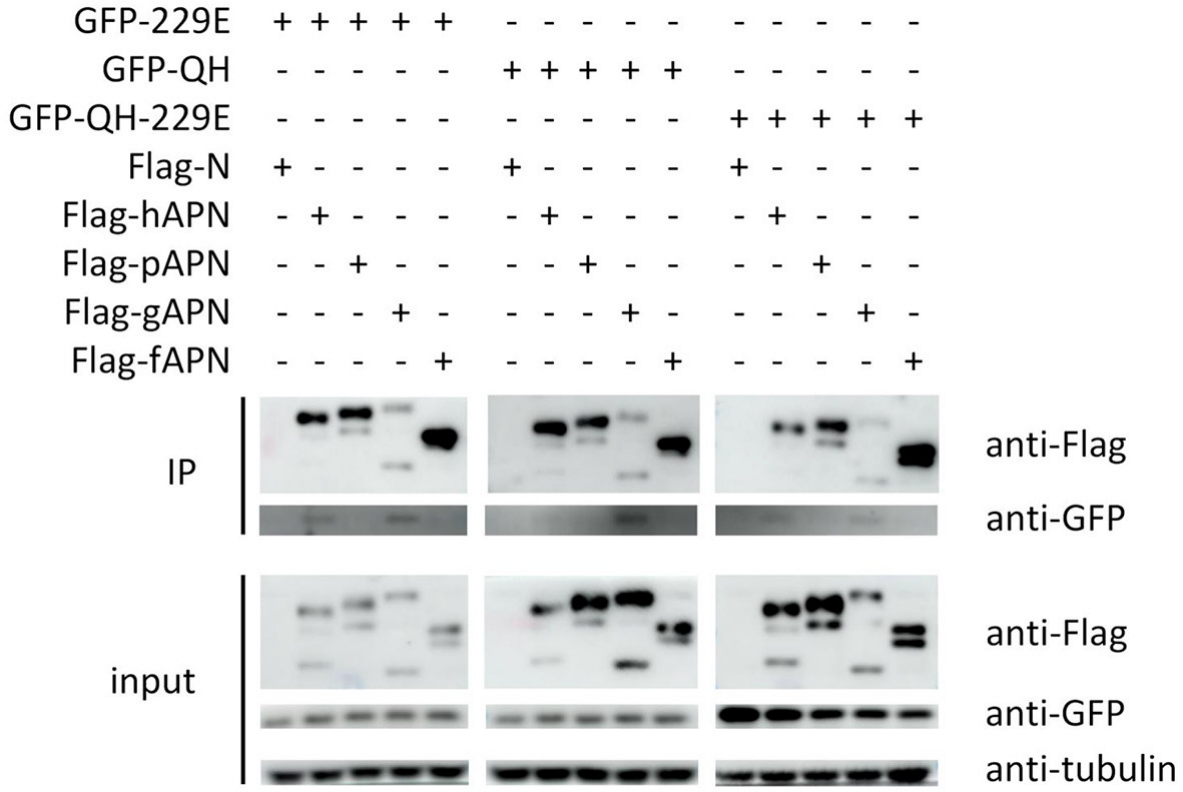
The co-immunoprecipitation (co-IP) results demonstrate that the RBD of the A/black-headed gull/Qinghai/2021–229E interacts with both human (hAPN) and gallus aminominopeptidase N (gAPN).

To validate the findings of molecular docking, co-immunoprecipitation experiments were conducted after co-transfecting cells with APN of host species and RBD of selected viruses, simultaneously. Our results demonstrated that 229E RBD interacts with both hAPN and gAPN, while BHG-QH-2021 RBD only interacts with gAPN. Most notably, BHG-QH-2021–229E RBD interacts with both hAPN and gAPN (Figure 11).

## 4 Discussion

Deltacoronavirus is the most recently discovered genus within the family Coronaviridae. With the advancements in sequencing technologies and extensive efforts, researchers have revealed that Deltacoronavirus do not adhere strictly to host specificity (Liang et al., 2019, 2023; Niu et al., 2021). Following reports of porcine *Coronavirus HKU15* infections in human in 2021, Deltacoronavirus has emerged as the third genus of Coronaviridae after Alphacoronavirus and Betacoronavirus, capable of infecting humans. Despite of these significant findings, the understanding of Deltacoronavirus beyond PDCoV remains limited, largely due to the scarcity of adaptable cell lines and the challenges associated with field sample collection. The lack of further insights presents a considerable obstacle in the prevention of emerging viral infectious diseases and the surveillance of viruses carried by wild animals.

Leveraging both existing data and our newly identified sequence, we conducted a comprehensive bioinformatics study on Deltacoronavirus. Geographically, although the number of full genome sequence in our collection did not exceed 50, Deltacoronavirus from wild bird have been detected on continents except Africa (Dong et al., 2007; Torres et al., 2017; Chen et al., 2018; Vibin et al., 2018; Barbosa et al., 2019; Wille et al., 2020). Considering the extensive migratory bird routes between Africa and Asia (Gu et al., 2021), in conjunction with reports from the northern Mediterranean coast and Middle East (Torres et al., 2017; Lau et al., 2018), we highly speculate that avian Deltacoronavirus has already achieved global distribution across all continents. We detected and analyzed a Deltacoronavirus strains derived from black-headed gull in Qinghai Lake, a critical node for the world's migratory birds populations. As reported by the local administration of Qinghai Lake National Nature Reserve, more than 33 species of 53,600 waterfowl were observed around Qinghai lake in spring of recent years. As shown by the collected information, waterfowl Anseriformes represent the major host type for Deltacoronaviruses with 30 reported cases of infection (Supplementary Table S1, Supplementary Figure S2), indicating that migratory behaviors may play a significant role in the transmission dynamics of Deltacoronaviruses. In addition, previously identified avian Deltacoronaviruse strains in Qinghai Province belong to *Putative species 3*, not *Putative species 4* which newly identified BHG-QH-2021 belong to, suggesting the diversity of avian Deltacoronaviruse in Qinghai Province.

In this study, our classification has expanded the strain numbers in the two species, *Coronavirus HKU15* and *Bulbul coronavirus HKU11*, and determined five putative species (*Putative species 1–5*), extending the 7 species of Deltacoronavirus classified by ICTV to 12 species, and clarified the classification relationships in Deltacoronavirus.

We found good confirmation of the species and genus relationships in the phylogenetic tree, where most of the viruses belonging to Buldecovirus are classified into the same subclade, while viruses belonging to Andecovirus and Herdecovirus are grouped into another subclade. The phylogenetic relation among the hosts infected by Deltacoronavirus was also analyzed and found to be divided into two clades, mammal and bird, linked by a dashed line in the host phylogenetic tree. Among the 13 species shown in Figure 4, only viruses in *Coronavirus HKU15* and *Putative species 3* infected both mammals and birds showing bird to mammal host cross-species transmission possibility. In terms of the diversity of infected hosts, although *Coronavirus HKU15* is the virus species with the largest number of identified sequences, only 2 species of birds and 2 species of mammals were reported to be infected by strains of this species (Woo et al., 2012; Durães-Carvalho et al., 2015; Chen et al., 2018; Domańska-Blicharz et al., 2019). In comparison, the viruses of *Putative species 4* were reported to infect 5 species of birds (Lau et al., 2018; Barbosa et al., 2019; Wille et al., 2019, 2020; Domańska-Blicharz et al., 2021; Chu et al., 2022; Marchenko et al., 2022), but not mammals, suggesting that the cross-species transmission of *Putative species 4* is more pronounced in birds. In additional, according to the collection information marked in Phylogenetic trees based on 7 conserved domains (Supplementary Figure S4), *Putative species 4* is characterized by inter-continental and inter-species distribution.

We performed a matrix analysis of strains and hosts based on the classification (Figure 4). The analysis showed that Deltacoronavirus has a broader host spectrum in birds compared to mammals, with only two species *Coronavirus HKU15* and *Putative species 3* reported to infect mammals so far. *Coronavirus HKU15* is the species PDCoV falls under. As mentioned above, *Common_magpie coronavirus (Putative species 5)* is a *Coronavirus HKU15*-related species and since *Coronavirus HKU15* could infect mammals, *Common_magpie coronavirus (Putative species 5)* poses a non-negligible risk of spillover to mammals. *Putative species 3* is a species detected from pikas and ground tits in the Qinghai Tibet Plateau, whose members have a consensus relationship with HKU17–6124 of *Coronavirus HKU15*, indicating a genomic association between *Putative species 3* and *Coronavirus HKU15*.

The S protein is a key functional protein involved in coronavirus invasion, pathogenicity and host tropism (Kumavath et al., 2021). In the phylogenetic analysis based on S protein (Supplementary Figure S5), we discovered a topological feature: HNU2 in *Common_magpie coronavirus (Putative species 5)* and White-eye coronavirus HKU16 were clustered together, while HNU1–2 and HNU1–1 were inserted into the topology of *Thrush coronavirus (Putative species 1)*. This finding is also reflected in the alignment of RBD amino acid sequence (Figure 8), indicating that *Common_magpie coronavirus (Putative species 5)* may adapt to host through recombination of S protein.

In the phylogenetic tree based on the 217-nt fragment of nsp12 gene of the Deltacoronavirus, we found that *Putative species 2* and *Munia coronavirus HKU13* were inserted into the branch of *Coronavirus HKU15*, separating the avian source viruses from mammalian source viruses in *Coronavirus HKU15*. This may indicate a more complex evolution relationship between *Coronavirus HKU15* and other strains of Deltacoronavirus. For other species, we observed the expected clustering as that in the phylogenetic trees based on genome, structural proteins and conserved domains. Overall, although shorter sequences were used in the phylogenetic analysis, the clustering of most branches is consistent with species classification. This method is effective and helpful for the identification of Deltacoronavirus species

In addition, in the phylogenetic analysis based on the 217-nt fragment, we found one branch that was not observed in the full-length genome-based analysis. The strain mw01_10 with full-length genome published failed to be classified into the same species with other strains with full-length genome, indicating that it may be a novel Deltacoronavirus. Genetically, the differentiation of this branch including mw01_10 strain and the strains from wild birds from Russia, Australia, and Poland only occurs after the subgenus Andecovirus (including *Wigeon coronavirus HKU20*) and the subgenus Herecovirus (including *Night-heron coronavirus HKU19*), indicating the intercontinental distribution and possibility of independent evolutionary origin of the branch.

The *Putative species 4*, to which BHG-QH-2021 belongs, has exhibited remarkable conservation throughout its evolutionary history, forming a stable branch in all phylogenetic trees based on genome sequences, conserved domains, structural proteins, and the 217-nt nsp12 fragment. The number of reported viruses within *Putative species 4* is second only to *Coronavirus HKU15*, highlighting its significance. Moreover, *Putative species 4* has been documented to infect hosts across four orders of wild birds, indicating its broad host range and ecological impact.

Recombination events are common in coronavirus. In 2018, Lau et al. reported a recombination event in the UAE-HKU27 strain with dut148cor1 and MW01_1o as major and minor parents, respectively (Lau et al., 2018). This finding is consistent with the major parents identified for BHG-QH-2021 in our study. Notably, in the classification process of our study, both UAE-HKU27 and BHG-QH-2021 were categorized into *Putative species 4*. When the recombinant relationship between *Putative species 4* strains and dut148cor1/MW01_1o was examined, we found that Deltacoronavirus strains of *Putative species 4* could also use dut148cor1 as their major parent, and MW01_1o as minor parent, suggesting that the strains in this species have similar recombination characteristics.

This study combines RBD sequence information and protein structure prediction to determine the interaction range of “229E related Deltacoronavirus RBD” as RBDs of *Putative species 2, Putative species 3, Putative species 4, Common-moorhen coronavirus HKU21, Wigeon coronavirus HKU20*, HKU17–6124 of *Coronavirus HKU15*, and HKU18-chu3, lrf178cor1, MW01_1o of *Deltacoronavirus sp*.. Different from those of *Bulbul coronavirus HKU11* strain HKU11–796, *Coronavirus HKU15* strain HKU17–6124, and *Munia coronavirus HKU13* strain HKU13–3514, which were able to utilize both gAPN and pAPN to invade host cells (Liang et al., 2023), the RBD of BHG-QH-2021 interacts with gallus APN (gAPN), but not with porcine APN (pAPN) and hAPN, as investigated by Co-IP experiment (Figure 11), a finding consistent with the binding affinity predicted by molecular docking calculations (Figure 10). Based on template-based modeling, we found that the best matching template for the RBD of BHG-QH-2021 was the RBD of 229E rather than that of PDCoV, indicating a greater structural similarity between the RBD of BHG-QH-2021 and 229E in their three-dimensional structure.

Next, we replaced the receptor-binding loop 1–2 segments of BHG-QH-2021 with that of 229E in the RBD of BHG-QH-2021 and found that the recombinant RBD could bind to both gAPN and hAPN (Figure 11), suggesting that the receptor-binding loop 1–2 is crucial for cross-species transmission of Deltacoronavirus.

In summary, to the best of our knowledge, this is the first report on the tracking and detection of coronavirus in wild birds in Qinghai Lake, an important node of the world migratory bird route. It is also the first record on *Putative species 4* in the Qinghai Tibet Plateau region. Analysis shows that the *Putative species 4* to which BHG-QH-2021 belongs to, is relatively conservative in phylogeny forming independent branch and is the second largest Deltacoronavirus species. The intraspecific phylogenetic characteristics of *Putative species 4* are more complex with infection capability of 5 orders of wildbirds. In terms of risk management, while the potential threat brought by the *Putative species 4* may be lower than that of mammal-related strains of Deltacoronavirus, structural analysis and experimental verification of the binding capacity of RBD of BHG-QH-2021 demonstrate its similarity to 229E, raising the possibility of its spillover to humans through recombination or mutation, an implication that we should not neglect.

## Data availability statement

The genome sequence generated in this study was submitted to the GenBank database (https://www.ncbi.nlm.nih.gov/genbank/), under accession number PP397109.

## Author contributions

YT: Data curation, Formal analysis, Methodology, Software, Validation, Visualization, Writing – original draft, Writing – review & editing. TY: Investigation, Methodology, Writing – review & editing. JW: Investigation, Methodology, Writing – review & editing. HZ: Methodology, Validation, Visualization, Writing – review & editing. YJ: Methodology, Writing – review & editing. XL: Methodology, Writing – review & editing. GeW: Methodology, Writing – review & editing. GuW: Methodology, Writing – review & editing. YH: Methodology, Writing – review & editing. CL: Writing – review & editing, Methodology. JZ: Resources, Writing – review & editing. LM: Conceptualization, Funding acquisition, Project administration, Resources, Supervision, Writing – review & editing. ML: Conceptualization, Data curation, Funding acquisition, Project administration, Resources, Supervision, Writing – review & editing.
