# Supplementary material for: Genetic characterization of the first Deltacoronavirus from wild birds around Qinghai Lake
Source: Front Microbiol. 2024 Jun 12;15:1423367. doi: 10.3389/fmicb.2024.1423367 (PMC11199898; doi:10.3389/fmicb.2024.1423367)
Supplement: Supplementary file 1 [file Data_Sheet_1.pdf]

Supplementary Material

1 Supplementary Figures and Tables

Supplementary Table 1. The full-length genomic strains used and the classification of the viruses

Accession	Species	Host order	Host genus	Host species	Isolate	Country	Continent	Year
EF584908		Carnivora	Prionailurus	Prionailurus_bengalensis	Guangxi/F230/2006	China	Asia	2006
EF584909		Carnivora	Melogale	Melogale_moschata	Guangxi/F247/2006	China	Asia	2006
EF584910		Carnivora	Melogale	Melogale_moschata	Guangxi/F250/2006	China	Asia	2006
EF584911		Carnivora	Melogale	Melogale_moschata	Guangxi/F247/2006	China	Asia	2006
EF584912		Carnivora	Melogale	Melogale_moschata	Guangxi/F250/2006	China	Asia	2006
EF584913		Carnivora	Melogale	Melogale_moschata	Guangxi/F247/2006	China	Asia	2006
EF584914		Carnivora	Melogale	Melogale_moschata	Guangxi/F250/2006	China	Asia	2006
FJ376619 (NC_011547)	Bulbul coronavirus HKU11	Passeriformes		Pycnonotus_sinensis	HKU11-934	China	Asia	2007
FJ376620	Bulbul coronavirus HKU11	Passeriformes		Pycnonotus_sinensis	HKU11-796	China	Asia	2007
FJ376621	Thrush coronavirus(Putative species 1)	Passeriformes	Turdus	Turdus_hortulorum	HKU12-600	China	Asia	2007
FJ376622 (NC_011550)	Munia coronavirus HKU13	Passeriformes	Lonchura	Lonchura_striata	HKU13-3514	China	Asia	2007
JQ065044 (NC_016991)	White-eye coronavirus HKU16	Passeriformes	Zosterops	Zosterops_japonicus	HKU16-6847	China	Asia	2007
JQ065045	Coronavirus HKU15	Passeriformes	Passer	Passer_montanus	HKU17-6124	China	Asia	2007
JQ065046	Deltacoronavirus sp.	Passeriformes		Copsychus_saularis	HKU18-chu3	China	Asia	2007
JQ065047 (NC_016994)	Night-heron coronavirus HKU19	Pelecaniformes	Nycticorax	Nycticorax_nycticorax	HKU19-6918	China	Asia	2007
JQ065049 (NC_016996)	Common-moorhen coronavirus HKU21	Gruiformes	Gallinula	Gallinula_chloropus	HKU21-8295	China	Asia	2007
JQ065048 (NC_016995)	Wigeon coronavirus HKU20	Anseriformes	Mareca	Mareca_penelope	HKU20-9243	China	Asia	2008
JQ065042	Coronavirus HKU15	Artiodactyla	Sus	Sus_scrofa	HKU15-44	China	Asia	2009
KP965434		Psittaciformes	Amazona	Amazona_vinacea	B04	Brazil	South America	2009
MK617495		Charadriiformes	Larus	Larus_canus	DeltaCoV/AvCoV/Common_tern/Poland/Mor340/2009	Poland	Europe	2009
MK617496		Galliformes		Marmota_spp.	DeltaCoV/AvCoV/Pheasant/Poland/MW378B/2009	Poland	Europe	2009
MK617497		Charadriiformes	Chroicocephalus	Chroicocephalus_ridibundus	DeltaCoV/AvCoV/black-headed_gull/Poland/Mor358/2/2011	Poland	Europe	2009
MK617514		Suliformes	Phasianus	Phasianus_colchicus	DeltaCoV/AvCoV/Cormorant/Poland/MW332/2009	Poland	Europe	2009

JQ065043 (NC_039208)	Coronavirus HKU15	Artiodactyla	Sus	Sus_scrofa	HKU15-155	China	Asia	2010
MK617515		Charadriiformes	Sterna	Sterna_hirundo	DeltaCoV/AvCoV/Common_gull/Poland/MW241/2011	Poland	Europe	2011
MK204388		Charadriiformes	Calidris	Calidris_acuminata	MW05	Australia	Oceania	2012
MK617498		Charadriiformes	Larus	Larus_canus	DeltaCoV/AvCoV/Common_gull/Poland/Mor17/4/2012	Poland	Europe	2012
KU321641		Charadriiformes	Rynchops	Rynchops_niger	PNLP115	Brazil	South America	2013
KU321643		Anseriformes	Anser	Anser_cygnoides	DPV_5	Brazil	South America	2013
KU321644		Anseriformes	Anser	Anser_cygnoides	DPV_10	Brazil	South America	2013
KU956099		Galliformes	Coturnix	Coturnix_coturnix	DCoV/quail/Brazil/2013 quail12F2 clone 1	Brazil	South America	2013
KU956100		Galliformes	Coturnix	Coturnix_coturnix	DCoV/quail/Brazil/2013 quail12F2 clone 2	Brazil	South America	2013
KU956101		Galliformes	Coturnix	Coturnix_coturnix	DCoV/quail/Brazil/2013 quail12F2 clone 4	Brazil	South America	2013
KU956102		Galliformes	Coturnix	Coturnix_coturnix	DCoV/quail/Brazil/2013 quail12F2 clone 6	Brazil	South America	2013
LC364342	Putative species 4	Falconiformes	Falco	Falco_peregrinus	UAE-HKU27	United Arab Emirates	Asia	2013
LC364343	Putative species 4	Otidiformes	Chlamydotis	Chlamydotis_macqueenii	UAE-HKU28	United Arab Emirates	Asia	2013
LC364345	Coronavirus HKU15	Galliformes	Coturnix	Coturnix_coturnix	UAE-HKU30 411F	United Arab Emirates	Asia	2013
MK617499		Charadriiformes	Larus	Larus_canus	DeltaCoV/AvCoV/Common_snipe/Poland/Mor78/25/2013	Poland	Europe	2013
MK617500		Charadriiformes	Larus	Larus_argentatus	DeltaCoV/AvCoV/black-headed_gull/Poland/Mor109/6/2013	Poland	Europe	2013
MK617501		Charadriiformes	Chroicocephalus	Chroicocephalus_ridibundus	DeltaCoV/AvCoV/black-headed_gull/Poland/Mor118pool2/2013	Poland	Europe	2013

LC364346	Coronavirus HKU15	Galliformes	Coturnix	Coturnix_coturnix	UAE-HKU30 1101F	United Arab Emirates	Asia	2014
MK617502		Charadriiformes	Chroicocephalus	Chroicocephalus_ridibundus	DeltaCoV/AvCoV/black-headed_gull/Poland/Mor001/36/2014	Poland	Europe	2014
MK617503		Charadriiformes	Larus	Larus_canus	DeltaCoV/AvCoV/Common_gull/Poland/P147/2/2014	Poland	Europe	2014
MK617505		Charadriiformes	Larus	Larus_canus	DeltaCoV/AvCoV/Common_gull/Poland/P151/4/2014	Poland	Europe	2014
MT025058		Sphenisciformes		Pygoscelis_papua	Antartic1		Antarctica	2014
MT993565	Deltacoronavirus sp.	Charadriiformes	Arenaria	Arenaria_interpres	MW01_1o	Australia	Oceania	2014
MW685622	Coronavirus HKU15	Primates	Homo	Homo_sapiens	Haiti/human/0081-4/2014	Haiti	North America	2014
KX077965		Galliformes	Coturnix	Coturnix_coturnix	quail/Italy/2015 quail01F1	Italy	Europe	2015
KX077966		Galliformes	Coturnix	Coturnix_coturnix	quail/Italy/2015 quail06F1	Italy	Europe	2015
KX077967		Galliformes	Coturnix	Coturnix_coturnix	quail/Italy/2015 quail21F1	Italy	Europe	2015
KX077968		Galliformes	Coturnix	Coturnix_coturnix	quail/Italy/2015 quail22F1	Italy	Europe	2015
KX077969		Galliformes	Coturnix	Coturnix_coturnix	quail/Italy/2015 quail38F1	Italy	Europe	2015
KX077970		Galliformes	Coturnix	Coturnix_coturnix	quail/Italy/2015 quail11F2	Italy	Europe	2015
KX077971		Galliformes	Coturnix	Coturnix_coturnix	quail/Italy/2015 quail30F4	Italy	Europe	2015
KX077972		Galliformes	Coturnix	Coturnix_coturnix	quail/Italy/2015 quail32F4	Italy	Europe	2015
MH532440	Coronavirus HKU15	Galliformes	Coturnix	Coturnix_coturnix	G032/2015	Poland	Europe	2015
MK617504		Charadriiformes	Chroicocephalus	Chroicocephalus_ridibundus	DeltaCoV/AvCoV/Black-headed_gull/Poland/P087/40/2015	Poland	Europe	2015
MK617506		Charadriiformes	Chroicocephalus	Chroicocephalus_ridibundus	DeltaCoV/AvCoV/Black-headed_gull/Poland/P149/10/2015	Poland	Europe	2015
MW685623	Coronavirus HKU15	Primates	Homo	Homo_sapiens	Haiti/human/0256-1/2015	Haiti	North America	2015
MW685624	Coronavirus HKU15	Primates	Homo	Homo_sapiens	Haiti/human/0329-4/2015	Haiti	North America	2015
MH013331		Anseriformes	Anas	Anas_superciliosa	Orf1b/550-2625/G001/AU/2016	Australia	Oceania	2016

MH013332		Anseriformes	Anas	Anas_superciliosa	Orf1a/2275-12924/G001/AU/2016	Australia	Oceania	2016
MH013333		Anseriformes	Anas	Anas_superciliosa	Orf1bstart/12921-14015/G001/AU/2016	Australia	Oceania	2016
MH013334		Anseriformes	Anas	Anas_superciliosa	Orf1b-NSP11-13/4-2622/G001/AU/2016	Australia	Oceania	2016
MH013335		Anseriformes	Anas	Anas_superciliosa	Orf1b-NSP11/111-2024/G001/AU/2016	Australia	Oceania	2016
MH013336		Anseriformes	Anas	Anas_superciliosa	EP/6426-6686/G001/AU/2016	Australia	Oceania	2016
MH013337		Anseriformes	Anas	Anas_superciliosa	SP/2623-6324/G001/AU/2016	Australia	Oceania	2016
MH013338		Anseriformes	Anas	Anas_superciliosa	MP/6569-7222/G001/AU/2016	Australia	Oceania	2016
MH013339		Anseriformes	Anas	Anas_superciliosa	NSP6/7226-7501/G001/AU/2016	Australia	Oceania	2016
MH090081		Charadriiformes	Arenaria	Arenaria_interpres	CoV-9567-2016/11/18-BB/TAS	Australia	Oceania	2016
MK617507		Charadriiformes	Larus	Larus_canus	isolate DeltaCoV/AvCoV/Common_gull/Poland/P016/9/2016	Poland	Europe	2016
MK617508		Charadriiformes	Chroicocephalus	Chroicocephalus_ridibundus	DeltaCoV/AvCoV/Black-headed_gull/Poland/P016/12/2016	Poland	Europe	2016
MK617509		Charadriiformes	Chroicocephalus	Chroicocephalus_ridibundus	DeltaCoV/AvCoV/Black-headed_gull/Poland/P040/8/2016	Poland	Europe	2016
MK617510		Charadriiformes	Chroicocephalus	Chroicocephalus_ridibundus	DeltaCoV/AvCoV/Hering_gull/Poland/P064/3/2016	Poland	Europe	2016
LC364344	Putative species 4	Columbiformes	Columba	Columba_livia	UAE-HKU29	United Arab Emirates	Asia	2017
MG812375	Coronavirus HKU15	Passeriformes	Passer	Passer_montanus	ISU690-4	United States (the)	North America	2017
MG812376	Coronavirus HKU15	Passeriformes	Passer	Passer_montanus	ISU690-7	United States (the)	North America	2017
MG812377	Coronavirus HKU15	Passeriformes	Passer	Passer_montanus	ISU42824	United States (the)	North America	2017
MG812378	Coronavirus HKU15	Passeriformes	Passer	Passer_montanus	ISU73347	United States (the)	North America	2017

MK617511		Charadriiformes	Gallinago	Gallinago_gallinago	DeltaCoV/AvCoV/Common_gull/Poland/P021/6/2017	Poland	Europe	2017
MK617512		Charadriiformes	Larus	Larus_canus	DeltaCoV/AvCoV/Common_gull/Poland/P114/1/2017	Poland	Europe	2017
MK617513		Charadriiformes	Chroicocephalus	Chroicocephalus_ridibundus	DeltaCoV/AvCoV/Black-headed_gull/Poland/P350/5/2017	Poland	Europe	2017
MN379902		Anseriformes	Spatula	Spatula_cyanoptera	Illinois2615	United States (the)	North America	2017
MN379903		Anseriformes	Spatula	Spatula_cyanoptera	Illinois2662	United States (the)	North America	2017
MN379904		Anseriformes	Spatula	Spatula_cyanoptera	Missouri3230	United States (the)	North America	2017
OM642545		Anseriformes	Anas	Anas_acuta	pintail/South Korea/SW9/2017	Korea (the Republic of)	Asia	2017
OQ535492	Putative species 4	Charadriiformes	Chroicocephalus	Chroicocephalus_ridibundus	BHGdCoV/Poland/P350/2017	Poland	Europe	2017
MT138104	Putative species 2	Passeriformes	Fringilla	Fringilla_montifringilla	brb027cor1	China	Asia	2018
MT138105	Putative species 2	Passeriformes	Fringilla	Fringilla_montifringilla	brb028cor1	China	Asia	2018
MT138106	Thrush coronavirus(Putative species 1)	Passeriformes	Turdus	Turdus_naumanni	dut148cor1	China	Asia	2018
MT138107	Deltacoronavirus sp.	Passeriformes	Carpodacus	Uragus_sibiricus	lrf178cor1	China	Asia	2018
MT138108	Putative species 2	Passeriformes	Emberiza	Emberiza_rustica	rub035cor1	China	Asia	2018
MT138109	Thrush coronavirus(Putative species 1)	Passeriformes	Turdus	Turdus_naumanni	thr147cor1	China	Asia	2018
MW345814	Common magpie coronavirus(Putative species 5)	Passeriformes	Pica	Pica_pica	HNU1-1	China	Asia	2018
MW345815	Common magpie coronavirus(Putative species 5)	Passeriformes	Pica	Pica_pica	HNU1-2	China	Asia	2018
MW345816	Common magpie coronavirus(Putative species 5)	Passeriformes	Pica	Pica_pica	HNU3	China	Asia	2018
MW349841	Common magpie coronavirus(Putative species 5)	Passeriformes	Pica	Pica_pica	HNU2	China	Asia	2018
OM642546		Anseriformes	Anas	Anas_crecca	teal/South Korea/SW24/2018	Korea (the Republic of)	Asia	2018
OM642547		Anseriformes	Anas	Anas_zonorhyncha	duck/South Korea/SW16/2018	Korea (the Republic of)	Asia	2018
OM642548		Anseriformes	Anas	Anas_zonorhyncha	duck/South Korea/SW13/2018	Korea (the Republic of)	Asia	2018
OQ535490	Putative species 4	Charadriiformes	Chroicocephalus	Chroicocephalus_ridibundus	BHGdCoV/Poland/P052/2018	Poland	Europe	2018

OQ535491	Putative species 4	Charadriiformes	Chroicocephalus	Chroicocephalus_ridibundus	BHGdCoV/Poland/P103/2018	Poland	Europe	2018
MT215336	Putative species 3	Passeriformes	Onychostruthus	Onychostruthus_taczanowskii	Montifringilla taczanowskii coronavirus N	China	Asia	2019
MT215337	Putative species 3	Rodentia	Marmota	Marmota_bobak	Montifringilla taczanowskii coronavirus HM	China	Asia	2019
OM642549		Anseriformes	Anas	Anas_zonorhyncha	duck/South Korea/SW14/2019	Korea (the Republic of)	Asia	2019
OM642550		Anseriformes	Anas	Anas_acuta	pintail/South Korea/SW18/2019	Korea (the Republic of)	Asia	2019
OM642551		Anseriformes	Anas	Anas_zonorhyncha	duck/South Korea/SW10/2019	Korea (the Republic of)	Asia	2019
OQ535493	Putative species 4	Charadriiformes	Larus	Larus_canus	CGdCoV/Poland/P005/2019	Poland	Europe	2019
OM480527		Passeriformes	Passer	Passer_montanus	FJ-NA5	China	Asia	2020
OM642552		Anseriformes	Anas	Anas_zonorhyncha	duck/South Korea/SW19/2020	Korea (the Republic of)	Asia	2020
OM642553		Anseriformes	Anas	Anas_crecca	teal/South Korea/SW21/2020	Korea (the Republic of)	Asia	2020
OM642554		Anseriformes	Anas	Anas_zonorhyncha	duck/South Korea/SW22/2020	Korea (the Republic of)	Asia	2020
OM642555		Anseriformes	Anas	Anas_platyrhynchos	Mallard/South Korea/SW11/2020	Korea (the Republic of)	Asia	2020
ON004916		Charadriiformes	Calidris	Calidris_ruficollis	ACoV/red-necked stint/Kamchatka/M20532/2020	Russian Federation (the)	Europe	2020
ON004917		Charadriiformes	Calidris	Calidris_ruficollis	ACoV/red-necked stint/Kamchatka/M20533/2020	Russian Federation (the)	Europe	2020
ON004918		Charadriiformes	Calidris	Calidris_alpina	ACoV/dunlin/Kamchatka/M20541/2020	Russian Federation (the)	Europe	2020
ON004919		Charadriiformes	Calidris	Calidris_alpina	ACoV/dunlin/Kamchatka/M20542/2020	Russian Federation (the)	Europe	2020
ON004920		Anseriformes	Mareca	Anas_strepera	ACoV/gadwall/Novosibirsk/M2127/2020	Russian Federation (the)	Europe	2020
ON004921		Anseriformes	Mareca	Anas_strepera	ACoV/gadwall/YaNAO-	Russian Federation (the)	Europe	2020

				Russia/M21342/2020			
ON004922	Anseriformes	Mareca	Anas_strepera	ACoV/gadwall/YaNAO-Russia/M21343/2020	Russian Federation (the)	Europe	2020
ON004923	Charadriiformes	Larus	Larus_heuglini	ACoV/siberian gull/YaNAO-Russia/M21362/2020	Russian Federation (the)	Europe	2020
ON004924	Charadriiformes	Larus	Larus_schistisagus	ACoV/slaty-backed gull/Kamchatka/M2326/2020	Russian Federation (the)	Europe	2020
ON004925	Charadriiformes	Calidris	Calidris_ruficollis	ACoV/red-necked stint/Kamchatka/M21282/2020	Russian Federation (the)	Europe	2020
ON004926	Charadriiformes	Calidris	Calidris_alpina	ACoV/dunlin/Kamchatka/M21292/2020	Russian Federation (the)	Europe	2020
ON004927	Charadriiformes	Calidris	Calidris_alpina	ACoV/dunlin/Kamchatka/M21293/2020	Russian Federation (the)	Europe	2020
ON004928	Charadriiformes	Calidris	Calidris_alpina	ACoV/dunlin/Kamchatka/M21302/2020	Russian Federation (the)	Europe	2020
ON004929	Charadriiformes	Calidris	Calidris_alpina	ACoV/dunlin/Kamchatka/M21303/2020	Russian Federation (the)	Europe	2020
ON004930	Charadriiformes	Calidris	Calidris_alpina	ACoV/dunlin/Kamchatka/M21311/2020	Russian Federation (the)	Europe	2020
ON605854	Anseriformes	Mareca	Anas_penelope	AvCoV/eurasion wigeon/YaNAO-Russia/M2140/2020	Russian Federation (the)	Europe	2020
ON605855	Passeriformes	Corvus	Corvus_cornix	AvCoV/hooded crow/YaNAO-Russia/M2139/2020	Russian Federation (the)	Europe	2020
ON605856	Charadriiformes	Stercorarius	Stercorarius_pomarinus	AvCoV/pomarine jaeger/YaNAO-Russia/M2138/2020	Russian Federation (the)	Europe	2020
OP355471	Pelecaniformes	Nycticorax	Nycticorax_nycticorax	AvCoV/black-crowned night-heron /Vietnam/M23411/2020	Viet Nam	Asia	2020
OP355472	Pelecaniformes	Nycticorax	Nycticorax_nycticorax	AvCoV/black-crowned night-heron /Vietnam/M2343/2020	Viet Nam	Asia	2020

OP355473		Pelecaniformes	Nycticorax	Nycticorax_nycticorax	AvCoV/black-crowned night-heron /Vietnam/M2344/2020	Viet Nam	Asia	2020
OP355474		Pelecaniformes	Nycticorax	Nycticorax_nycticorax	AvCoV/black-crowned night-heron /Vietnam/M2345/2020	Viet Nam	Asia	2020
OP355475		Suliformes	Phalacrocorax	Phalacrocorax_sulcirostris	AvCoV/indian cormorant/Vietnam /M2347/2020	Viet Nam	Asia	2020
OP355476		Suliformes	Phalacrocorax	Phalacrocorax_sulcirostris	AvCoV/indian cormorant/Vietnam /M2348/2020	Viet Nam	Asia	2020
OP355477		Pelecaniformes	Nycticorax	Nycticorax_nycticorax	AvCoV/black-crowned night-heron/Vietnam/M2349/2020	Viet Nam	Asia	2020
OP355478		Pelecaniformes	Egretta	Egretta_garzetta	AvCoV/little egret /Vietnam/M2340/2020	Viet Nam	Asia	2020
OP355479		Pelecaniformes	Nycticorax	Nycticorax_nycticorax	AvCoV/black-crowned night-heron /Vietnam/M23414/2020	Viet Nam	Asia	2020
OP355480		Pelecaniformes	Nycticorax	Nycticorax_nycticorax	AvCoV/black-crowned night-heron /Vietnam/M2342/2020	Viet Nam	Asia	2020
OP355481		Pelecaniformes	Nycticorax	Nycticorax_nycticorax	AvCoV/black-crowned night-heron/Vietnam/M23491/2020	Viet Nam	Asia	2020
OP355482		Pelecaniformes	Egretta	Egretta_garzetta	AvCoV/little egret /Vietnam/M23511/2020	Viet Nam	Asia	2020
OP355483		Pelecaniformes	Nycticorax	Nycticorax_nycticorax	AvCoV/black-crowned night-heron /Vietnam/M23452/2020	Viet Nam	Asia	2020
OL311150	Putative species 4	Charadriiformes	Chroicocephalus	Chroicocephalus_ridibundus	HNU4-1	China	Asia	2021
OL311151	Putative species 4	Charadriiformes	Chroicocephalus	Chroicocephalus_ridibundus	HNU4-2	China	Asia	2021

OL311152	Putative species 4	Charadriiformes	Chroicocephalus	Chroicocephalus_ridibundus	HNU4-3	China	Asia	2021
PP397109	Putative species 4	Charadriiformes	Chroicocephalus	Chroicocephalus_ridibundus	A/black-headed_gull/Qinghai/2021[1]	China	Asia	2021
OQ107245		Passeriformes	Passer	Passer_montanus	BIME-WS31	China	Asia	2022
OR344774					A220778	Brazil	South America	2022
OR344775					A220801	Brazil	South America	2022
OR344776					A220810	Brazil	South America	2022

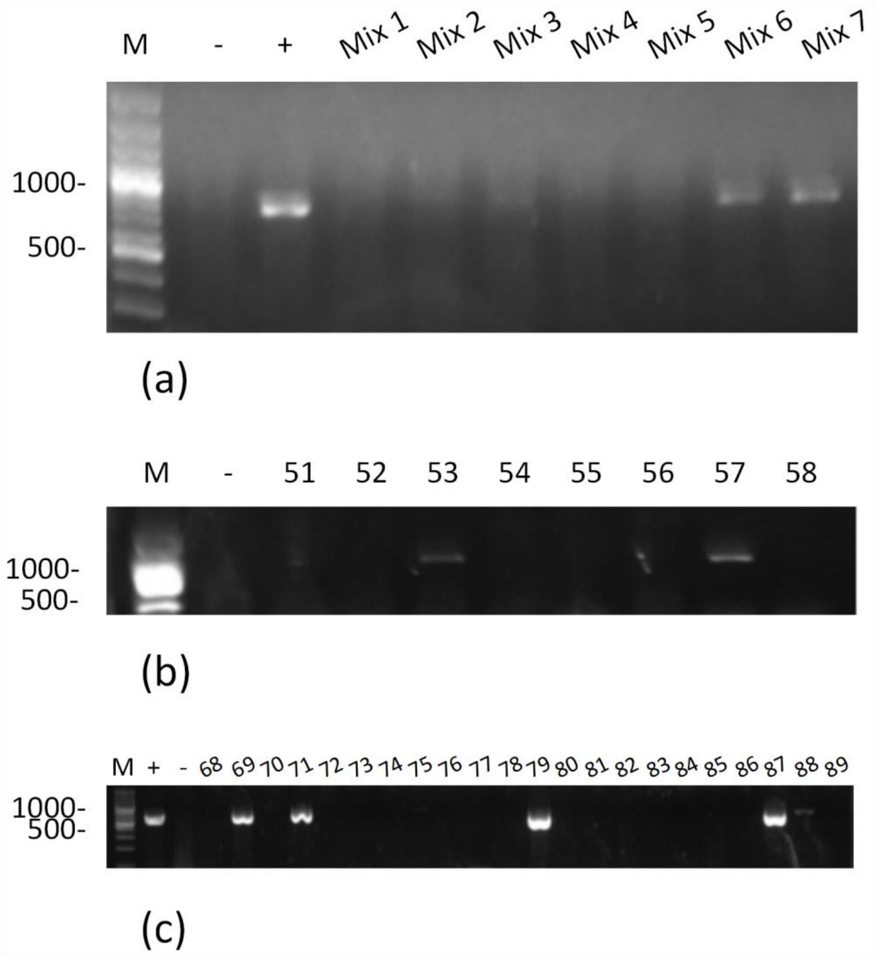

Supplementary Figure S1. Detection of Deltacoronavirus in fecal samples of wild bird by RT-PCR. (a) Detection of pooled samples. Individual samples (n) were pooled into one pool (n=10, with the last pool n=7) for initial screening. (b) Detection of individual samples from the positive pool (pool 6). (c) Detection of another collection of field samples stored in Trizol collected from the same regions. Only part of the samples including positives were shown.

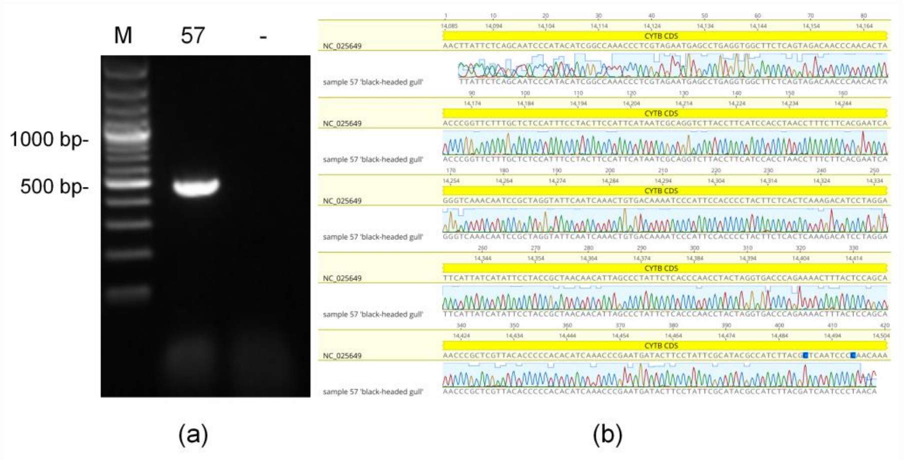

Supplementary Figure S2. Identification of host species of sample 57. (a) Amplification of Cytochrome b (*Cytb*) gene fragment by RT-PCR. (b) Comparison of *Cytb* gene fragment sequence of sample 57 with that of black-headed_gull (NC_025649).

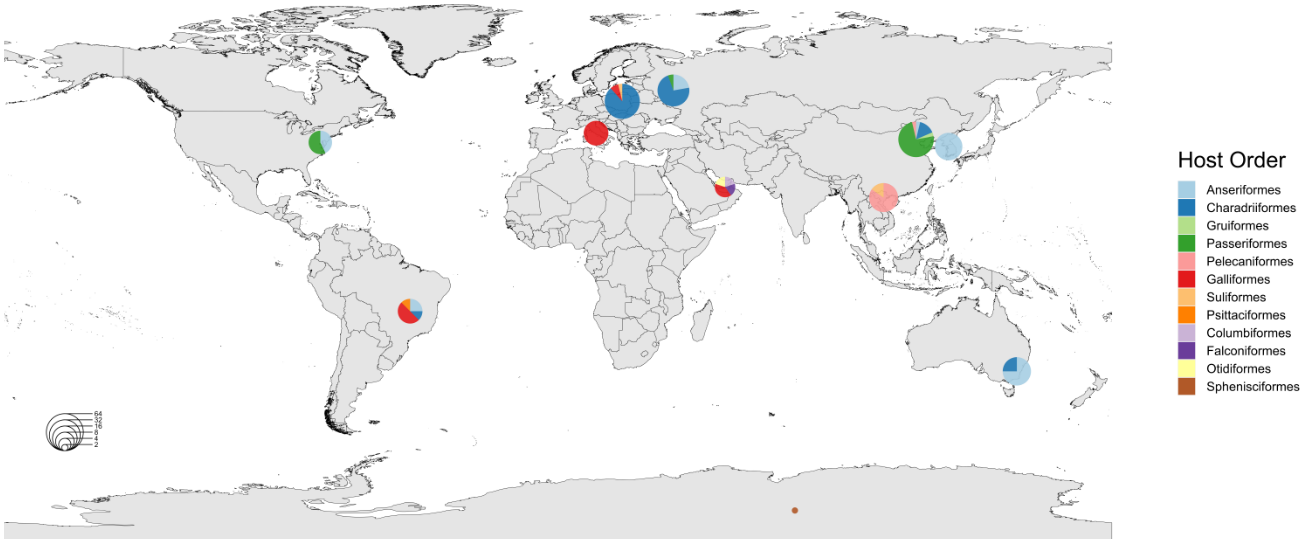

Supplementary Figure S3. Distribution of 136 avian Deltacoronaviruse strains with clear information on geographic location and host species. Strain numbers corresponding to slice sizes were shown on the lower left. Host species indicated by different colors were listed on the right.

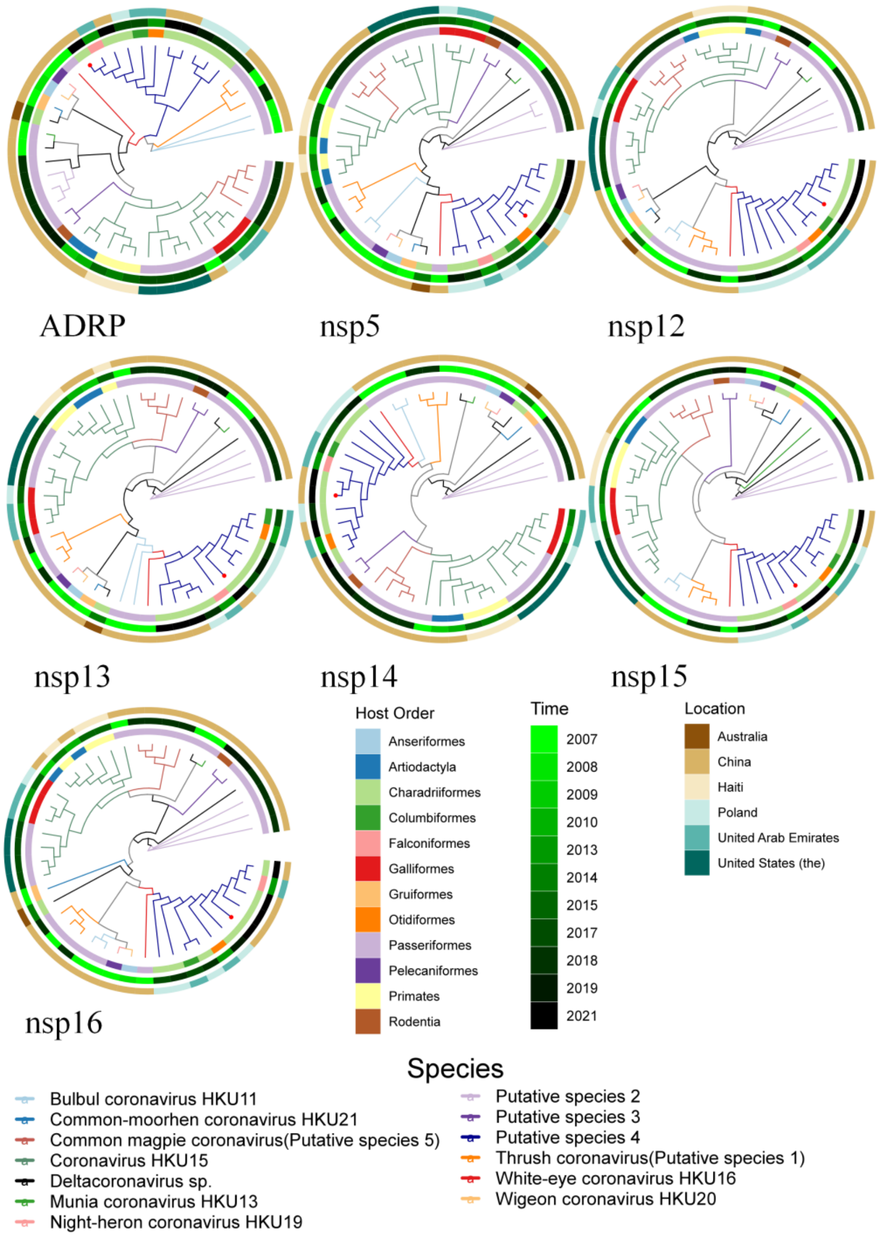

Supplementary Figure S4. Maximum likelihood phylogenetic trees based on the conserved domains in the Orf1ab region of 46 full-length strains of Deltacoronavirus. Branch lengths are omitted to emphasize topological relationships between branches. Viral species are classified using different colors, with the novel strain A/black-headed_gull/Qinghai/2021 detected in this study represented by

a red triangle. Data on host orders, collection time, and collection location are annotated in successive concentric rings from inner to outer.

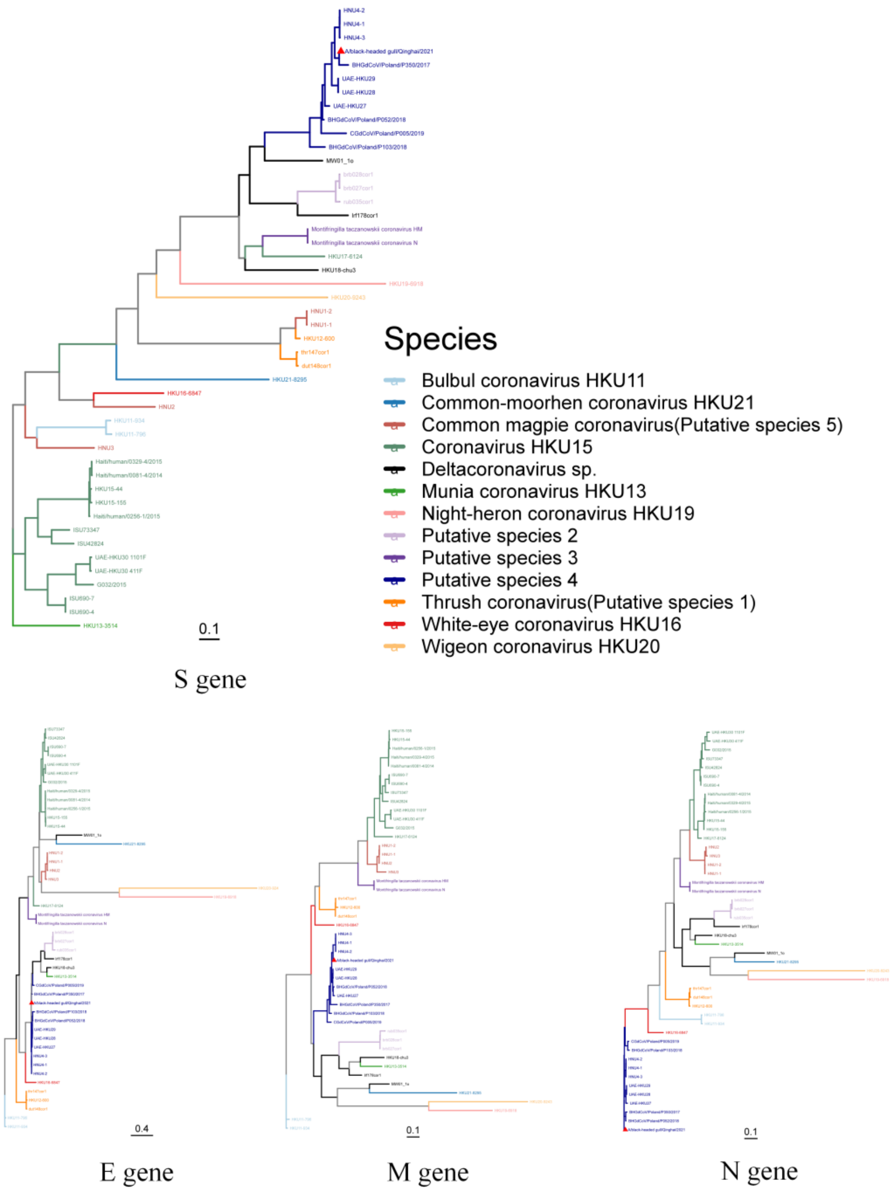

Supplementary Figure S5. Maximum likelihood phylogenetic trees of structural proteins in 46 full-length strains of Deltacoronavirus. Viral species are classified using different colors. The novel strain A/black-headed_gull/Qinghai/2021 detected in this study is represented by a red triangle.
